# A glimpse into novel acylations and their emerging role in regulating cancer metastasis

**DOI:** 10.1007/s00018-023-05104-z

**Published:** 2024-02-05

**Authors:** Huifang Shi, Weigang Cui, Yan Qin, Lei Chen, Tao Yu, Jie Lv

**Affiliations:** 1 Clinical Laboratory, The Rizhao People’s Hospital Affiliated to Jining Medical University, No. 126 Taian Road, Rizhao, 276826 Shandong China; 2 Central Laboratory, The Rizhao People’s Hospital Affiliated to Jining Medical University, No. 126 Taian Road, Rizhao, 276826 Shandong China; 3 Center for Regenerative Medicine, Institute for Translational Medicine, The Affiliated Hospital of Qingdao University, No. 16 Jiangsu Road, Qingdao, 266000 China; 4 Department of Cardiac Ultrasound, The Affiliated Hospital of Qingdao University, No. 16 Jiangsu Road, Qingdao, 266000 China

**Keywords:** Metastasis, Epigenetic modification, Novel acylation modification, Pathological implication, Therapy, Diagnostic biomarkers

## Abstract

Metastatic cancer is a major cause of cancer-related mortality; however, the complex regulation process remains to be further elucidated. A large amount of preliminary investigations focus on the role of epigenetic mechanisms in cancer metastasis. Notably, the posttranslational modifications were found to be critically involved in malignancy, thus attracting considerable attention. Beyond acetylation, novel forms of acylation have been recently identified following advances in mass spectrometry, proteomics technologies, and bioinformatics, such as propionylation, butyrylation, malonylation, succinylation, crotonylation, 2-hydroxyisobutyrylation, lactylation, among others. These novel acylations play pivotal roles in regulating different aspects of energy mechanism and mediating signal transduction by covalently modifying histone or nonhistone proteins. Furthermore, these acylations and their modifying enzymes show promise regarding the diagnosis and treatment of tumors, especially tumor metastasis. Here, we comprehensively review the identification and characterization of 11 novel acylations, and the corresponding modifying enzymes, highlighting their significance for tumor metastasis. We also focus on their potential application as clinical therapeutic targets and diagnostic predictors, discussing the current obstacles and future research prospects.

## Introduction

Cancer is a leading cause of mortality worldwide. Despite many studies having focused on its diagnosis and treatment, > 10 million cancer-related mortalities occur annually [1]. Failed cancer therapy is mainly due to metastasis, a hallmark of cancer, causing approximately 90% of cancer-related deaths [2]. Metastasis is a multifactorial cascade wherein disseminated primary tumor cells invade the local extracellular matrix, intravasate into the circulation, escape immune surveillance, and subsequently colonize distal sites. Exploring the key regulators of tumor metastasis is widely regarded as an important solution to alleviate the high mortality rate of cancer. During metastasis, multiple regulatory elements, including both genetic and epigenetic factors, are implicated in this cascade. Genes that generally mediate metastasis are grouped into three classes [3]: the first correlates with the acquired motility of cancer cells, angiogenesis, and immune escape and are thus called metastasis initiation genes; the second—metastasis progression genes—are essential for malignant tumor cells to permeate capillary vessels and survive in secondary metastatic organs; the third group—metastasis virulence genes—are required to colonize metastatic target organs [4, 5]. Along with genetic engagement, epigenetically organized programs are now being implicated as key drivers of successful metastasis. For instance, increasing evidence has revealed that no known recurrent mutations present during pancreatic ductal carcinoma (PDA) metastasis, highlighting the significance of epigenetic regulation in cancer metastasis [6]. Therefore, epigenetic factors are pivotal in the metastatic cascade, rendering them potential diagnostics and therapeutics targets.

Epigenetics include DNA methylation, post-translational modifications (PTMs), and noncoding RNAs, which introduce phenotypic changes without altering the gene sequence. PTMs alter the structure of the existing proteins, thereby regulating their biological function. Excluding genetic diversity, PTMs increase the functional complexity of proteomics. Acylation is a widespread PTM with key functions in most physiological systems. Acetylation was the first cognate lysine acylation discovered to be implicated in multiple cellular functions; with advances in high-resolution mass spectrometry (MS) technology, additional acylation mechanisms have been identified beyond acetylation, making acylation a ‘hot spot’ for epigenetic modifications.

These novel acylations are catalyzed by both enzymatic and nonenzymatic mechanisms. In enzymatic catalysis, acyltransferases and deacylases facilitate the transfer and removal of acyl groups. Emerging evidence suggests that dysregulation of these novel acylations strongly correlates with numerous diseases, including cancer, neurological disorders, metabolic diseases, etc. Some inhibitors targeting novel acylation modifications have been used clinically, yielding promising results; e.g., inhibitors targeting acyltransferases p300 and CREB-binding protein (p300/CBP) have been under clinical evaluation in patients with hematologic malignancies or advanced and drug-resistant solid tumors [7]. Chidamide, a histone deacetylase (HDAC) inhibitor (HDACi), was clinically approved for patients with peripheral T-cell lymphomas in China in 2017 [8]; therefore, revealing the roles of novel acylation modifications in refractory diseases is critical for effective therapeutic strategies.

**Fig. 1:**
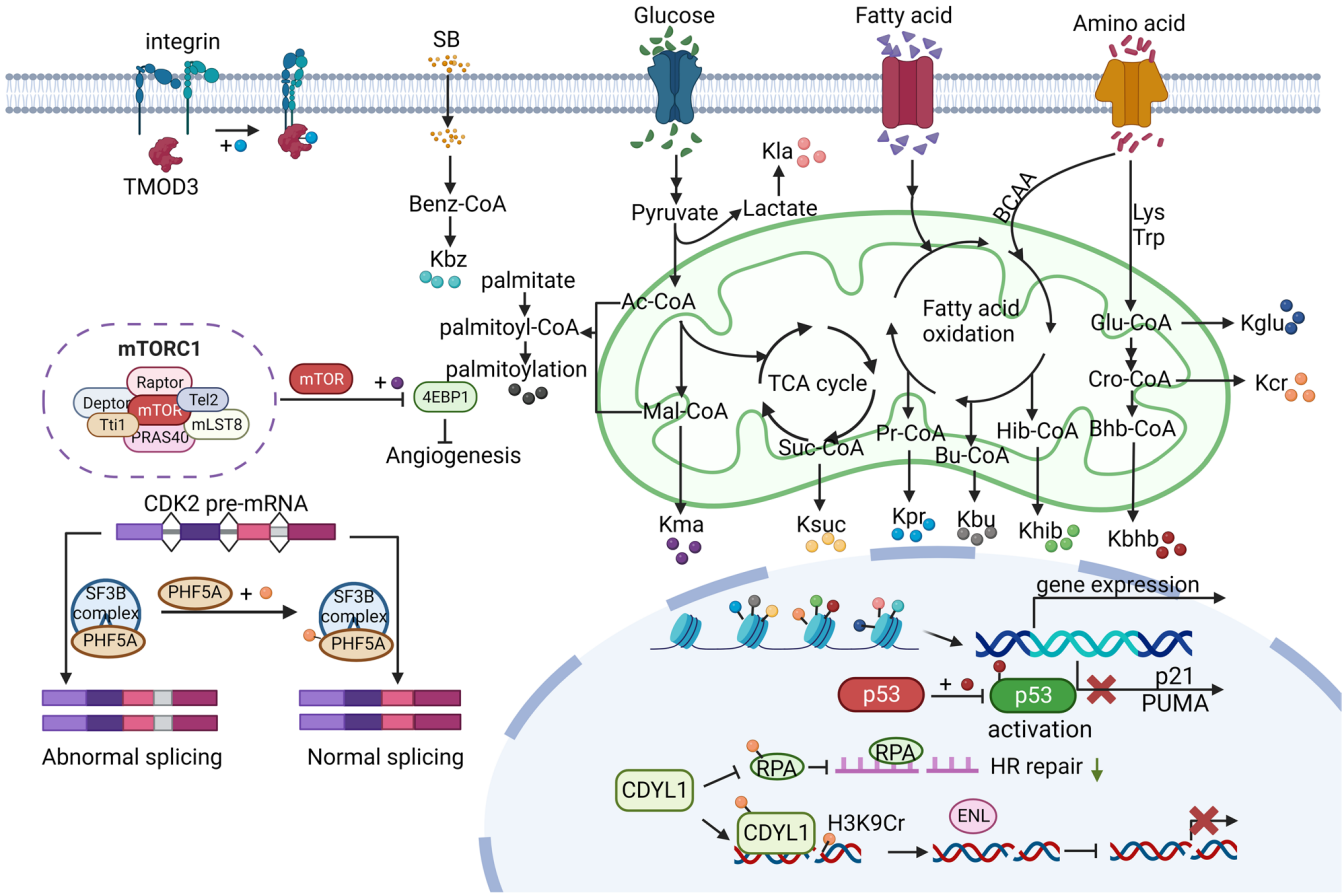
The main functions and metabolic origins of novel acylations. In the mitochondria, multiple metabolic pathways generate acyl-CoA, which provide acylation groups to covalently modify proteins. In the nucleus, histone acylations preferentially accumulate at the promoters of active genes, and participate in the transcription initiation of active genes. Kcr regulates the homologous recombination DNA repair, and promotes gene silencing. The Kbhb of p53 decrease p53 enzymatic activity, and thus attenuating the transcriptional expression of p21 and PUMA. In the cytoplasm, various regulatory functions are involved, including modulating kinase activity and pre-mRNA splicing. (Created with BioRender.com.)

Currently, the research on novel acylations is enormous and growing, and a considerable number of reports have reviewed these acylations and their regulatory mechanisms. For instance, Sabari et al. summarized the regulatory function of acylations on gene expression through metabolism [9], and the recent progress in nonhistone protein acylations was highlighted by Shang et al. [10]. The extensive roles of acylations in histone and nonhistone were also surmised in health and disease, particularly their functional roles in tumorigenesis and tumor microenvironment [11–13]. Nevertheless, extensive research has suggested a close correlation between novel acylation modifications and tumor metastasis. Indeed, acylation-associated regulatory mechanisms receive considerable attention, in particular regarding tumor metastasis. Hence, in this review, the significant roles of novel acylations in regulating tumor metastasis are discussed, and their clinical significance in the diagnosis and treatment of cancer metastasis is highlighted. Overall, we review the identification and characterization of 11 novel acylations (Fig. 1), and comprehensively describe their regulatory enzymes (Table 1). In addition, the emerging, diverse role of novel acylations in tumor metastasis is emphasized, and its therapeutic potential is discussed. An in-depth exploration of novel acylations of target proteins and their regulatory elements in tumor metastasis might provide meaningful developments for effective disease control and offer a broad clinical application in tumor therapy.

**Table 1:** Characteristics and functions of noval acylations

Acylations	Writers	Erasers	Readers	Donors	Function and mechanism	References
Propionylation	p300/CBP; GNAT family(HATs, P/CAF, GCN5L2); MYST family(MOF, MOZ, KAT6 and HBO1)	SIRT1; SIRT3; SIRT5;	Bromodomain (BRD4);YEATS domain	Propionyl-CoA; propionate; odd-chain fatty acid and amino acid	Transcription activation; protein stability; energy metabolism	[23–29]
Butyrylation	p300/CBP; P/CAF; GCN5; HBO1	SIRT1-3; SIRT6; SIRT7	Bromodomains (CECR2, BRD9, TAF1, BRD4)	Butyryl-CoA; β-oxidation of fatty acids	Transcription activation; adipogenesis and fat metabolism; spermiogenesis	[34–38]
Malonylation	N/A	SIRT5; Sir2	N/A	Malonyl-CoA	Inflammation; angiogenesis; metabolic disorders	[45–49]
Succinylation	α-KGDH complex; KAT2A; HAT1; CPT1A	SIRT; SIRT7; CobB	N/A	Succinyl-CoA	Transcription activation; metabolism modulation (especially mitochondrial metabolic pathways)	[53, 56–59]
Crotonylation	p300; MYST family (human MOF and its yeast homolog Esa1)	SIRT1-3; HDAC1; HDAC3	YEATS domains (Yaf9, ENL, AF9, Taf14 and Sas5); DPF domains (MOZ and DPF2)	Crotonyl-CoA	Translation initiation; RNA splicing; DNA damage and repair; cell cycle	[61, 67, 69, 70, 71]
Glutarylation	KAT2A; α-KADH	SIRT5; SIRT7	N/A	Glutaryl-CoA	Sperm motility; transcription regulation; oxidative damage	[74, 76, 77]
2-Hydroxyisobutyrylation	p300; Tip60; Esa1p	HDAC1-3; Rpd3p; Hos3p; CobB	N/A	2-Hydroxyisobutyryl-CoA	Transcription activation; biosynthesis of amino acids, and glycolysis/gluconeogenesis; sperm motility	[78, 79, 81, 83]
β-Hydroxybutyrylation	p300/CBP	HDAC1-3; SIRT3	N/A	β-Hydroxybutyryl-CoA; β-hydroxybutyrate	Gene transcription; modulates redox homeostasis in CD8 + T_mem_ cells; lipolytic and ketogenic metabolism; antagonize aortic endothelial injury in diabetes	[85, 88–90]
Benzoylation	HBO1; p300/CBP; SAGA complex	SIRT2; Hst2	DPF and YEATS family; bromodomain (Sth1)	Benzoyl-CoA	Transcription activation; glycolysis/gluconeogenesis, ribosome biogenesis, and rRNA processing	[93–95]
Lactylation	p300	HDAC1-3; SIRT1-3	N/A	Lactyl-CoA (theoretically)	Gene expression; macrophage reprogramming; increase endothelium permeability; neural excitation and social stress; microglial dysfunction in AD; RNA splicing	[97, 100, 101–104, 106]
Palmitoylation	DHHC1–23	APT1–2	N/A	Palmitoyl-CoA	Protein localization, and membrane trafficking; Protein stability; enzymatic activity; secretion	[109–114, 116]

*N/A* not available

## The identification and characterization of novel acylation

### Propionylation

Lysine propionylation (Kpr) was first identified and validated in 2007 via high-performance liquid chromatography-tandem mass spectrometry (HPLC/MS/MS) analysis in a proteomics study [14]. Acetyl-CoA is a donor for protein acetylation, while propionyl-CoA is the substrate for lysine propionylation, catalyzed by acyltransferases derived from the catabolism of odd-chain fatty acids and amino acids. To date, no propionylation-specific nonacetyl acyltransferases have been identified. Multiple histone acetyltransferases (HATs) were identified with an expanded repertoire of acyltransferase activities. The first detected enzyme to catalyze the propionylation of core histones H3 and H4 in mammalian cells was p300/CBP [15]. Nonhistones, such as p53 and bacterial propionyl-CoA synthetase, can also be propionylated by p300/CBP; however, the catalytic activity is higher for p300 than for CBP [16]. Interestingly, p300 and CBP could also be autopropionylated at lysine residues in a manner similar to their autoacetylation [17]. The general control nonrepressible 5 (Gcn5)-related N-acetyltransferase (GNAT) family, including HATs, p300/CREB-binding protein-associated protein (P/CAF), and general control of amino acid synthesis yeast homolog like 2 (GCN5L2), exhibited effective propionyltransferase activity [18]. In addition, the recent studies found that MYST family members of lysine acetyltransferases (KATs), including MOF, MOZ, KAT6, and HBO1, displayed both propionyltransferase and acetyltransferase activities [19]. Consistent with deacetylation, lysine depropionylation is also catalyzed by HDACs, including sirtuin (SIRT)-1, SIRT3, and SIRT5, which were shown to reduce propionylation in an NAD(+)-dependent manner [17, 20]. Furthermore, the bromodomain of bromodomain-containing protein 4 (BRD4) and the Yaf9, ENL, AF9, Taf14, Sas5 (YEATS) domain are recognized as “readers” of propionylation; BRD4 and the YEATS domain exhibit a lower and higher binding affinity for Kpr than Kac, respectively [21, 22].

Propionylation influences cellular and organelle functions by mediating DNA binding affinity, protein stability, and enzyme activity; e.g., histone H3 propionylation was detected to preferentially accumulate at the promoters of active genes and was recognized as a mark of chromatin activation [23, 24]. Xu et al. reported that tropomodulin-3 propionylation determines the stability of integrin αIIbβ3 thus underlying integrin-mediated cell spreading [25]. Moreover, propionyl-CoA derived from amino acids and fatty acid catabolism regulated cell differentiation and aging, with histone propionylation enriched at the specific differentiation sites of the MyoD gene [26]. Research advances proved that propionylation regulates the function of subcellular components and is closely associated with energy metabolism. In patients with genetically defective propionyl-CoA carboxylase, the pathologic accumulation of propionyl-CoA increased the level of protein propionylation in liver cells and decreased mitochondrial respiration; nevertheless, mitochondrial respiration did not decrease with propionate in C2C12 myotubes [27]. This inconsistency may be attributed to the differences in propionyl-CoA metabolism between liver and muscle myotubes, as propionate exposure increased propionyl-CoA levels in liver cells, while in muscle, propionyl-CoA is mainly derived from the amino acid metabolism but not propionate. Meanwhile, it was revealed that propionyl-CoA carboxylase deficiency in *Caenorhabditis elegans* resulted in reduced lifespan through disordered energy metabolism, comprising mitochondrial OXPHOS capacity reduction, oxidative stress increase, and distal tricarboxylic acid (TCA) cycle flux inhibition [28]. In line with these findings, lysine propionylation was also found to mediate energy metabolism in *Aeromonas hydrophila*, while site-specific propionylation modifications negatively regulate malate dehydrogenase metabolic activity [29]. Thus, propionylation plays a crucial role in modulating gene expression, maintaining protein stability, and participating in energy metabolism.

### Butyrylation

Lysine butyrylation (Kbu) was identified along with propionylation in 2007 [14]. Butyryl-CoA is a metabolic intermediate product of the β-oxidation of fatty acids; it is structurally similar to propionyl-CoA and acetyl-CoA, with only one or two CH2 differences. Both propionylation and butyrylation share most of their catalytic enzymes, including p300/CBP, P/CAF, GCN5, and HBO1. Notably, although some enzymes share common regulatory pathways for propionylation and butyrylation, P/CAF and GCN5 exhibit less enzymatic activity for butyryl-CoA than for propionyl-CoA [18]. Similarly, deacetylase SIRT family members showed different selectivities for catalytic groups: SIRT2 and SIRT6 prefer debutyrylation to deacetylation, while SIRT1 and SIRT3 exhibit stronger removal capacity for acetyl than butyryl groups, and SIRT7 displays a similar capacity for removing both acetyl and butyryl groups [30]. Contrastingly, a previous study reported that SIRT1, SIRT2, and SIRT3 tended to remove short-chain acyl groups [31]. It is speculated that whether SIRT2 binds to nucleosomes or peptides determines its preference for acyl groups. This hypothesis could be interpreted by analyzing the cocrystalline structure of SIRT2 and nucleosomes. In addition, the CECR2, BRD9, and TAF1 bromodomains were confirmed to read the long butyryl group; still, most human bromodomains only tend to bind the relatively short acetyl and propionyl groups [32]. A recent study revealed that the functional genomic distribution of bromodomain factors is regulated by the histone acetylation/longer-chain acylation(s) ratio derived from metabolism. The increased nonacetyl acylation (crotonylation or butyrylation) to acetylation ratio of H4K5 weakens the chromatin interaction, enhancing nuclear mobility and availability in a BRD4 bromodomain-dependent manner to bind to the transcription initiation sites of active genes [33].

Considerable evidence confirmed that butyrylation is an effective booster modulating the metabolic regulation of gene expression. Histone butyrylation was reported to be enriched in the promoters of proadipogenic genes during adipogenesis. To further confirm that the catalytic activity of p300 in adipogenesis is for butyrylation (not acetylation), LTK-14A, a semisynthetic derivative of garcinol, was administrated. This specifically suppressed histone butyrylation without acetylation, and results demonstrated that LTK-14A treatment significantly inhibited the butyrylation of H4K5 and downregulated the expression of proadipogenic genes, thus culminating in abolished adipogenesis [34]. Nevertheless, it was demonstrated that H3K9 butyrylation in human cells and mouse hearts is most enriched in mice fed a fat-free rather than high-fat diet, revealing the negative correlation with high-fat diets [35]. Interestingly, diet-related butyrylation was demonstrated to be sex-dependent, with starvation promoting the butyrylation of histone lysine in the liver cells in a gender-selective manner (only detected in males) [36]. Thus, increased histone butyrylation during starvation reinforces the concept of histone modification, linking the interaction between metabolism and epigenetics. Moreover, an interplay between histone acetylation and butyrylation in gene transcriptional regulation during sperm cell differentiation has also been clarified; the dynamic changes between acetylation and butyrylation in histone H4K5 and K8 at gene promoters are recognized as hallmarks of highly active genes [37], and the occurrence of H4K5 butyrylation and acetylation have different starting points during spermiogenesis [38]. Therefore, these data improve our understanding of the functional implications of butyrylation for gene promoter activity.

### Malonylation

Lysine malonylation (Kma), an evolutionarily conserved and dynamic PTM in bacteria and mammals, was first detected in 2011 [39]. In 2012, histone Kma was investigated in HeLa cells and *Saccharomyces cerevisiae*, and the modulation sites were revealed [40]. Thereafter, Kma was discovered to be widely distributed; e.g., 4042 Kma sites were detected on 1426 mouse liver proteins, and 4943 Kma sites were identified on 1822 proteins in human fibroblasts [41]. Consistent with other acylations, Kma is theoretically modulated by KATs and HADCs; however, reports regarding their catalytic enzymes are limited. Among the known components of these mechanisms, the protein Kma is driven by the concentration of malonyl-CoA that is controlled by the activities of acetyl-Coenzyme A carboxylase (ACC), propionyl-CoA carboxylase (PCC), fatty acid synthase (FASN) and malonyl-CoA decarboxylase (MCD). By testing the demalonylation activity of all 18 HDACs, including HDAC1–11 and SIRT1–7, only SIRT5 exhibited prominent lysine demalonylation activity [39, 42]. Sir2, a yeast sirtuin homolog, displaced demalonylation activity in yeast [43]; however, whether typical acyltransferases (p300/CREB, HATs, and HBO1) participate in catalyzing Kma remains to be investigated.

Kma is linked to diverse physiological processes, including inflammation, angiogenesis, and metabolic disorders. Macrophages generally undergo classical activation (M1) and alternative activation (M2), which play different functions in inflammation and tissue homeostasis. Numerous studies have recently focused on the roles of metabolic reprogramming in determining the functional responses of macrophages [44]. Specifically, malonylation was detected following the activation of bone marrow-derived macrophages, which depends on ACC1-induced malonyl-CoA production. Particularly, the malonylation of GAPDH on K213, an essential enzyme in glycolysis, caused its dissociation from mRNA in response to lipopolysaccharides (LPS), thereby facilitating the translation of several inflammatory mRNAs [45]. In addition to macrophages, emerging evidence has revealed the involvement of malonylation in the metabolic reprogramming of endothelial cells. A study on FASN knockdown in endothelial cells showed that FASN silencing elevated malonyl-CoA levels, leading to mTOR malonylation at K1218. Interestingly, mTOR malonylation inhibited the kinase activity of mTOR complex 1, thus hampering angiogenesis and neovascularization [46]. Furthermore, various research has emphasized the significance of malonylation in metabolism; e.g., Kma proteins are involved in multiple metabolic processes, including RNA degradation, energy metabolism, and the synthesis of various secondary metabolites [47]. Increased malonylation in cells causes impaired mitochondrial function, fatty acid oxidation, and glucose metabolism [41, 48]; therefore, the increased Kma introduced by SIRT5 deficiency was observed to decrease basal mitochondrial respiration and reduce the rate of glycolysis in primary chondrocytes [49]. Overall, these data highlight the critical role of malonylation in disease, indicating its potential as a target for clinical treatment.

### Succinylation

Lysine succinylation (Ksuc) was first identified in isocitrate dehydrogenase, hydroxymethyltransferase, and GAPDH in 2011 and was verified through three independent methods, including MS/MS, HPLC coelution, and western blot analysis [50]. Compared with Kac, Ksuc causes a two-unit charge shift in modified lysine residues, inducing greater mass changes and converting the side chain from positively to negatively charged. Succinyl-CoA, a nonenzymatic and enzymatic donor of Ksuc, is derived from the TCA cycle or amino acid metabolism. Although succinyl-CoA governs the rate of Ksuc depending on pH and concentration [51], many catalytic enzymes regulate the addition and removal of succinyl groups from proteins; e.g., α-ketoglutarate dehydrogenase complex controls Ksuc owing to the E2k succinyltransferase activity [52], while KAT2A acts as a succinyltransferase, binding to this complex and succinylating histone H3 on K79 nearby the transcription initiation sites of genes [53]. In HepG2 cancer cells, HAT1 was also found to regulate the Ksuc of both histones and nonhistones (e.g., histone H3 on K122 and PGAM1 on K99) [54]. Surprisingly, carnitine palmitoyltransferase 1A (CPT1A) was also verified to exert lysine succinyltransferase activity using succinyl-CoA as a substrate [55]. In mammalian cells, both SIRT5 and SIRT7 displayed desuccinylation activity; SIRT7 removes histone Ksuc (which functionally promotes chromatin condensation and double-strand break [DSB] repair), while SIRT5 exerts desuccinylase activity in all cell compartments [56]. Interestingly, SIRT5-mediated desuccinylation of targets is protein-selective, and not all succinylated lysines are affected by SIRT5; e.g., SIRT5 can only catalyze 8/32 mitochondrial CPT1A-dependent Ksuc-modulated proteins [55]. In addition, the SIRT2-like enzyme CobB was identified as a prokaryotic bifunctional enzyme that can remove both a succinyl and acetyl group in *E. coli* [57]*.* These findings support that multiple different acylations may share the same catalytic enzyme.

Consistent with many other novel acylations, Ksuc has a substantial impact on gene expression and energy metabolism. By evaluating the KEGG pathway, annotating the GO analysis, and interpreting the Pfam domain in *E. coli*, Ksuc substrates were found to be closely associated with translation-associated events and participate in ribosome pathways [58]. In human U251 glioblastoma (GBM) and HEK-293 cells, KAT2A depletion expectedly decreased the level of Ksuc in specific gene promoter sites [53]; SIRT7 is recruited to DNA DSBs dependent on poly(ADP-ribose) polymerase 1 (PARP1), thereby promoting DSB repair and chromatin condensation [56]. Therefore, Ksuc is instrumental in the regulation of gene expression. Besides gene expression regulation, studies have shown that many succinylated proteins participate in modulating metabolism, especially mitochondrial metabolic pathways that are likely to affect their enzymatic function; e.g., high-glucose treatment increased-succinylated proteins are enriched in metabolic pathways [57]. The strong association between Ksuc and cellular energy status implies a significant role in energy metabolism. Notably, the effect of glucose is more pronounced in Ksuc than in Kac, with the high glucose enhancing the abundance of succinylated compared with acetylated proteins [57]. Hence, it may be useful to integrate the response of Ksuc to metabolic challenges. Succinylated substrates are extremely concentrated in mitochondrial metabolic pathways; SIRT5 deficiency leads to substantially increased succinate dehydrogenase (SDH) and pyruvate dehydrogenase (PDH) complex activity, implying the critical engagement Ksuc in the TCA cycle and electron transport chain [59].

### Crotonylation

Innovative research resulted in the first report of lysine crotonylation (Kcr) in 2011. The genomic localization and unique structure of histone crotonylation determine its functional and mechanical difference from histone Kac. Initially, CBP, p300, and HDAC6 exhibited minmal effect on crotonylation and decrotonylation in 293 T cells [60]. Improvements in detection technology illustrated that p300 possesses histone crotonyltransferase activity in cell-free assays that are sensitive to changes in crotonyl-CoA concentration [61]. Furthermore, the catalytic activity of p300 is greater for histone Kcr than Kac, and p300-catalyzed histone Kcr was found to directly stimulate gene transcription. Following the discovery of p300, MYST family proteins, MOF, and its yeast homolog Esa1 were also reported to transfer crotonyl groups to lysine residues [62]. In addition, SIRT family members (including SIRT1–3) were discovered to recognize the histone H3K4Cr mark, while only SIRT3 possesses relatively tight and selective binding to H3K4Cr [63]. Interestingly, it was demonstrated that class I HDACs (HDAC1 and HDAC3), which have distinct site-specificity from SIRT1, facilitate decrotonylation; e.g., the decrotonylation of HDAC1 is active for H3K4, H3K9, H3K18, H3K23, H4K8, and H4K12, while SIRT1 is inactive against H3K23 and H4K12 [64]. YEATS domain proteins are a family of histone acylation readers that identify a series of short-chain acylations with a predilection for crotonylation [65]. Coincidentally, double plant homeodomain finger (PHD) finger (DPF) domains of human MOZ and DPF2 (or KAT6A and BAF45d, respectively) also adapted to extensive histone lysine acylations with a preference for Kcr [66].

Kcr participates in various biological processes, including translation initiation, RNA splicing, the cell cycle, DNA damage, and DNA repair. Similar to butyrylation and propionylation characterized as marks of gene activation histone Kcr is also considered a reliable indicator of active promoters [61]. Notably, p300-mediated histone crotonylation was reported to be stronger than histone acetylation for stimulating gene transcription [61]. PHD finger protein 5A (PHF5A) is a highly conserved protein found to regulate pre-mRNA splicing. A study reported that decrotonylation of PHF5A in K25 reduced the cyclin-dependent kinase 2 (CDK2) expression through abnormal splicing induced by retained introns [67]. Chromodomain Y-like (CDYL) was shown to negatively regulate histone Kcr as a crotonyl-CoA hydratase [68]. A large-scale proteomics analysis for protein Kcr has revealed that CDYL negatively regulates replication protein A1 (RPA1) Kcr, and impairs its role in homologous recombination DNA repair. Mechanically, crotonylated RPA1 enhances its interaction with single-stranded DNA and resection machinery components [69]. Furthermore, the counteraction of crotonylated CDYL1 with H3K9Cr at DSB regions releases the transcription elongation factor ENL, promoting gene silencing. This discovery poses a confusing question concerning the functional correlation between DSB-induced silencing and homologous recombination repair [70]. In addition, the precise attachment of astral microtubules to the lateral cell cortex depends on TIP60-regulated end-binding protein 1 (EB1) Kcr, which helps to dynamically link their interaction [71]. These findings suggest therapeutic strategies via targeting crotonylation in the clinic.

### Glutarylation

Lysine glutarylation (Kglu) was first detected and comprehensively validated in 2014 through immunoblot and MS, combined with chemical and biochemical methods [72]. Kglu involves adding a five-carbon glutaryl group to the lysine residue, shifting the charge from negative to positive [73]. Like succinyl-CoA, glutaryl-CoA can directly induce the Kglu in a nonenzymatic manner; this is influenced by the glutaryl-CoA concentration and the ratio of glutaryl-CoA to other CoAs [9, 72]. Recently, Kglu was found to be enzymatically accomplished in histones. Accompanied by the identification of Kglu, SIRT5 was verified to catalyze the enzymatic removal of lysine glutaryl residues [72]; subsequently, SIRT7 and KAT2A coupled with α-KADH were designated deglutarylase and glutaryltransferase, respectively, to dynamically stabilize H4K91glu [74].

Growing evidence confirms the strong association between Kglu and metabolism. Proteome-wide screening in mouse liver revealed that the mitochondrial proteins and metabolic enzymes were highly enriched with Kglu modification [72]; moreover, 73 Kglu sites were identified on 37 mitochondrial proteins from the brain and found to be involved in various metabolic degradation pathways [75]. Considering the enrichment of glutarylation in mitochondria and the significance of mitochondria for sperm motility, Kglu was examined in sperm; the results demonstrated that the average Kglu level in sperm was significantly lower in asthenozoospermic than normozoospermic males and positively correlated with progressive motility [76]. These findings prove that Kglu plays a coordinating role in metabolism, thereby affecting sperm motility. In addition, multiple histone and nonhistone proteins were glutarylated, revealing their significant roles in transcription regulation and oxidative damage. Generally, many acylation modifications are considered modulators that regulate chromatin structure and dynamics. Undoubtedly, Kglu was found to play an indispensable role during various DNA-associated chromatin remodification processes; e.g., it was reported that Kglu at histone H4K91 destabilized the nucleosome and influenced the stable structure of chromatins, thereby leading to the global upregulation of transcription [74]. Zhou et al. demonstrated that glucose-6-phosphate dehydrogenase deglutarylation and isocitrate dehydrogenase 2 desuccinylation activated NADPH-producing enzymes in a SIRT5-dependent manner, thereby regulating cellular NADPH homeostasis and protecting the cells from oxidative damage [77]. Therefore, Kglu modulates the regulation of gene expression and metabolism and may be highly correlated with multiple biological functions.

### 2-Hydroxyisobutyrylation

Lysine 2-hydroxyisobutyrylation (Khib) was discovered through MS/MS and HPLC coelution experiments in 2014 [78]. Histone Khib was discovered in mammalian cells as an evolutionarily conserved modification in eukaryotic cells, including mouse embryonic fibroblast and HeLa cells, as well as in *Drosophila* and yeast cells [78]. Khib originates from 2-hydroxyisobutyrate and 2-hydroxyisobutyrate-derived 2-hydroxyisobutyryl-CoA, the cofactor engaged in enzymatic reactions. Similar to other acylations, Khib is regulated by enzymes that catalyze the addition and removal of this modification. Consistent with the acyltransferase activity in acetylation and crotonylation, p300 positively regulated the Khib levels in histones and nonhistones of mammalian cells, manifesting with different substrate selectivity toward Khib and Kac [79]. Tip60 and its yeast homolog, Esa1p, were identified as “writers” , allowing the 2-hydroxyisobutyryl group into the catalytic pocket to catalyze H4Khib [80]. In addition, HDAC2 and HDAC3 exhibit deacylase activity toward Khib, while HDAC1 only slightly affected the total histone Khib level both in vivo and in vitro [81]*.* In *S. cerevisiae*, H4K8hib is induced by the histone lysine deacetylases Hos3p and Rpd3p [81]; moreover, CobB can catalyze lysine deacylation for both Khib and Ksuc in prokaryotes [82].

According to existing reports, Khib is significantly associated with gene transcription and metabolism. Corresponding with the activation marks, p300-catalyzed histone Khib directly activated the gene transcription of p53 [79]. Transcription-activated genes in mouse meiotic and postmeiotic cells were detected with H4K8hib, which is considered a novel indicator of gene transcription activity [78]. Nevertheless, H4K8hib modification was diminished in low-glucose conditions [81], underlying the potential of Khib in metabolism-related pathways. KEGG pathway enrichment analysis identified that p300-mediated Khib was selectively enriched in the biosynthesis of amino acids, carbon metabolism, and glycolysis/gluconeogenesis. Remarkably, 5/10 catalyzed enzymes involved in glycolysis were p300-dependent 2-hydroxyisobutylations in HCT116 cells, and Khib-modulated glycolytic enzymes were essential for regulating glycolysis in response to nutrient availability [79, 81]. According to proteome analyses, alpha/gamma-enolase 1/2 (ENO1/2), phosphoglycerate kinase 1 (PGK1), fructose-bisphosphate aldolase A (ALDOA), ATP-dependent 6-phosphofructokinase muscle (PFKM) type, and glucose-6-phosphate isomerase (GPI) were all 2-hydroxyisobutyrylated. Specifically, it was confirmed that the p300-mediated Khib of PFKM and ENO1 activated their glycolytic enzymatic activity. In addition, Khib was found to be enriched in human sperm tails and inversely correlated with their progressive motility [83]. Moreover, ribosome, proteasome, and spliceosome pathway-associated proteins were involved in Khib marks in HeLa cells and *Proteus mirabilis* [80, 84]. These investigations reveal the close link between Khib and metabolism-related gene expression.

### β-Hydroxybutyrylation

Lysine β-hydroxybutyrylation (Kbhb) was initially identified in 2016 as a novel histone modification derived from β-hydroxybutyrate [85]. β-hydroxybutyrate, together with acetoacetate and acetone, are components of ketone bodies and provide energy to the brain and heart during starvation. Ketone bodies are produced predominantly by acetyl-CoA derived from fatty acid oxidation (FAO) and glucose oxidation. The cellular concentration of β-hydroxybutyrate regulates histone Kbhb; therefore, prolonged fasting-associated β-hydroxybutyrate determines the level of histone Kbhb. Cellular β-hydroxybutyrate generates β-hydroxybutyryl-CoA as the cofactor for Kbhb through short-chain-CoA synthetase [85]. P300/CBP is a confirmed histone Kbhb acyl transferase that catalyzes H4K8bhb and H3K18bhb in cells. Notably, p300 catalytic activity was stronger at histone Kbhb than corresponding Kac sites; contrastingly, PCAF and GCN5 did not significantly alter H4K8bhb and H3K18bhb levels [86]. Furthermore, the simultaneous knockdown of HDAC1 with HDAC2 enhanced Kbhb levels in HeLa and HEK293 cells; no remarkable changes in histone Kbhb sites were observed by individually overexpressing HDAC1-3, suggesting dynamic regulation via multiple HDACs [86]. Additionally, SIRT3 exhibited class-selectivity regarding the de-β-hydroxybutyrylation of histone H3 (not H4) both in vitro and in cells; HDAC3 did not display class-selectivity [87].

The prominent biological function of histone Kbhb is gene transcription regulation. Various metabolism-related gene activations are associated with H3K9bhb following starvation; the top five metabolic pathways are amino acid catabolism, circadian rhythm, redox balance, the PPAR signaling pathway, and oxidative phosphorylation [85]. Specifically, the histone H3K9bhb of Foxo1 and Ppargc1a upregulates the expression of these genes, thereby regulating Pck1 expression, which directs glycogen metabolism and thus modulates redox homeostasis in CD8^+^ T_mem_ cells [88]. In addition, in small intestine crypts during fasting, H3K9bhb accumulates in the proximal promoter regions of essential genes that correlate with ketogenic and lipolytic metabolic pathways [89]. Furthermore, H3K9bhb elevation caused by β-hydroxybutyrate facilitates the production of VEGF, thus antagonizing aortic endothelial injury in diabetic rats [90]. Overall, histone H3K9bhb is an essential element that regulates metabolism-related gene expression. In addition to histone Kbhb, nonhistone Kbhb simultaneously exhibits discriminative effects on cellular modulation; e.g., β-hydroxybutyrate-mediated p53 Kbhb attenuated p53 activity and reduced p21 and PUMA expression (downstream of p53) [91]. High-throughput proteomic analysis of Kbhb from the livers of starved mice revealed that Kbhb has a comprehensive cellular impact; multiple critical metabolic enzymes were seriously β-hydroxybutyrylated, and were mainly enriched in amino acid, detoxification, fatty acid, and one-carbon metabolic pathways [92]. Overall, histone and nonhistone Kbhb highly correlate with metabolism, thus paving the foundation for metabolic disease therapy.

### Benzoylation

Lysine benzoylation (Kbz) was first discovered in core histones of HepG2 and RAW cells in 2018 [93]. It was demonstrated that Kbz is an evolutionarily highly conserved histone modification that can be identified from mammalian and *Drosophila* cells. Interestingly, differing from histone Kcr and Kac (which is widespread in N-terminal and other regions), Kbz modifications mainly occur on N-terminal tails, differentiating Kbz from histone Kcr and Kac in chromatin modulation. Consistent with other acyl-CoAs, benzoyl-CoA, a core intermediate of aromatic growth substrate degradation in intestinal flora and bacteria, is produced in response to sodium benzoate availability in mammalian cells and serves as the donor for Kbz. Following HDAC screening, SIRT2 exhibited direct debenzoylation activity in vivo and in vitro [93]*.* Thereafter, both HBO1 and p300/CBP were identified as catalysts of Kbz; Kbz remained unaffected by Tip60, GCN5, HAT1, MOF, and PCAF overexpression [94]. Notably, the catalytic activity of HBO1 was weaker for Kac than Kbz [94]. According to this study, benzoyl-CoA and acetyl-CoA may competitively bind to the active regions of HBO1, thereby affecting the substrate selectivity of HBO1 and causing different functional outcomes. By systematically screening Hbz modulators in yeast, the Spt-Ada-Gcn5 acetyltransferase (SAGA) complex was recognized as the writer and NAD^+^-dependent HDAC Hst2 as the eraser of histone Kbz, respectively [95]. As Kbz readers, DPF and YEATS family proteins (not BRD domains) were found to bind benzoyl groups in humans, with YEATS2 demonstrating a preference for Kbz [96].

Analogously, Kbz was observed to be associated with multiple biological functions, including gene transcription and cell metabolic pathways. Histone Kbz was discovered to be enriched in gene promoters, and the Kbz levels positively correlated with gene expression at transcription start sites (TSSs) [93]. Tan et al. analyzed that HBO1 catalyzed Kbz to participate in various cellular physiological and pathological processes, including chromatin remodeling and transcription regulation [94]. Despite reports that emphasize the importance of Kbz in regulating gene expression, current proteome-wide screening identified that the metabolic pathways, including glycolysis/gluconeogenesis, ribosome biogenesis, and rRNA processing pathways, were enriched in Kbz-modified nonhistone proteins [95].

### Lactylation

Lysine lactylation (Kla) was predicted and identified in 2019, which was inspired from the discovery of the other histone acylations as their common origin from metabolic pathways [97]. Kla can be stimulated by lactate that was a metabolite in aerobic glycolysis, and was widely recognized as an energy source [97]. Enzyme activities involved in glycolysis and mitochondrial metabolism, such as PDH and lactate dehydrogenase (LDH), modulate histone Kla by regulating endogenous lactate levels [97]. Theoretically, there should be a lactyl-CoA that involved in possessing a class of substrates to directly provide a lactyl group to lysine residues, while no evidence comfirmed the existence of lactyl-CoA in vivo. However, it was estimated that the acetyl-CoA synthase 2 (ACSS2), might generate lactyl-CoA since the highly coincidence of catalytic enzymes between lactylation and acetylation, and ACSS2 has been verified to produce acetyl-CoA in acetylation [98]. A recent study showed that p300 might potentially catalyze the histone lactylation, although its indirect effect cannot be excluded right now [97]. Afterwards, HDAC1-3 and SIRT1-3 were determined with delactylase activity in vitro, and HDAC1-3 were confirmed to have the de-L-lactylase activity in cells [99].

Based on the discovery history of Kla, it is not difficult to speculate the function of Kla with gene expression and metabolism reprogramming. Studies showed that lactate-induced histone Kla facilitate the switch of macrophages from a pro-inflammatory phenotype to a homeostatic phenotype [97, 100]. Specific wound healing genes were expressed at later time points in response to lipopolysaccharide (LPS), interferon (IFN)-γ or bacterial stimulation, which was well in line with the production of intracellular lactate and the leve of histone Kla [97]. Moreover, reparative gene transcription was boosted by histone Kla remotely and early in circulating and bone marrow located monocytes after myocardial infarction (MI) [101]. In macrophages during polymicrobial sepsis, lactate was found to catalyze lactylation and acetylation of high-mobility group box-1 (HMGB1). The HMGB1 modified by both Kla and Kac is secreted in a manner of exosome, which increased the permeability of endothelium [95]. Besides monocyte-macrophages, increased Kla level was also detected in brain cells. One study showed that the social stress and neural excitation determined the level of Kla, in parallel with lactate level in brain [102]. Recently, another study demonstrated that the level of H4K12la was rised in Aβ plaque-adjacent microglia of Alzheimer's disease (AD), which is accumulated at the promoter regions of glycolytic genes and increases glycolytic activity, thus form a positive feedback loop that exacerbates microglial dysfunction [103]. Furthermore, abnormal metabolism in nonsmall cell lung cancer was verified to be attributed to Kla as well, as showed that histone lactylation upregulate the expression of TCA cycle enzymes, thus attenuating glycolysis and maintaining mitochondrial homeostasis [103, 104]. Interestingly, Kla was recently found to be enriched in the spliceosomal pathway as well [105]. Given the potential correlation between metabolism and reparative genes expression, lactylation is now recognized as the linker to hands metabolic and immune regulation [106, 107].

### Palmitoylation

Different from the above-mentioned short-chain acylation modification to lysine residue, protein palmitoylation is a covalent modification of protein with 16-carbon palmitic acid to cysteine residue. Palmitoylation generally refers to S-palmitoylation. Although palmitoylation was discovered in the early 1970s, little is known about its regulatory mechanisms and functions. The development of novel proteomics technology and cell-imaging techniques accelerated the pace of research in this field. Palmitoylation and de-palmitoylation of target proteins reversibly cycle in response to upstream signals as a result of the unstable nature of thioester bonds. As the donor of palmitoylation, palmitoyl-CoA can be formed from palmitate catalyzed by cytosol acyl-coA synthetase (ACS). There was evidence of both enzymatic and nonenzymatic pathways for palmitoylation, with the enzymatic mechanism occupy a dominant role. Palmitoylation was catalyzed by palmitoyl S-acyltransferases (PATs), which contain a zinc finger Asp-His-His-Cys (ZDHHC) domain that is necessary for their catalytic activity, and depalmitoylation was carried out by acylprotein thioesterases (APTs) [108].

Because of the attachment of saturated fatty acid to target proteins, palmitoylation strengthens the hydrophobicity of proteins. In addition, it also plays essential roles in regulating protein localization, stability, enzymatic activity, membrane trafficking and secretion. For instance, the palmitoylation of H/N-Ras determines their plasma-membrane localization in a site-specific manner [108]; DHHC13-mediated EGFR palmitoylation is essential for its plasma-membrane localization [109]. CD36 palmitoylation is necessary for its processing, trafficking and fatty acid uptake activity [110]. Furthermore, palmitoylation is essential for keeping the plasma-membrane localization of G protein-coupled receptors (GPCRs) and GLUT1 [111, 112]. In addition, a study showed that the palmitoylation of PD-L1 in its cytoplasmic domain enhance its stability by hampering its ubiquitination [113]. In contrast, a recent study found that DHHC12-mediated palmitoylation of leucine-rich repeat and pyrin-domain-containing 3 (NLRP3) facilitate its lysosomal degradation through the chaperone-mediated autophagy (CMA) pathway [114]. Studies have also found that APT1 deficiency-induced defective depalmitoylation causes insulin hypersecretion thus resulting in β-cell exhaustion [115].

## Roles of novel acylations in tumor metastases

Since novel acylation modifications influence multiple aspects of physiological and pathological processes, including gene expression, metabolism, and protein stability and activity, they are proposed to play a critical role during tumor metastasis. Several studies have linked alternative novel acylations with various malignant tumor metastases. In this section, we mainly review the associations between these novel acylations and metastasis.

### Head and neck cancer

Head and neck cancer is one of the most common cancers worldwide, accounting for about 3% of all malignancies [116]. The relationship between Kac and head and neck carcinoma is well-established and includes activation of immunosuppression-related molecule expression (such as programmed cell death 1 ligand 1 [PD-L1] and galectin-9) [117] and epithelial-mesenchymal transition (EMT) [118], thus affecting angiogenesis [119]. In oral squamous cell carcinoma (OSCC), multiple proteins with Khib upregulation were detected; this correlated with the actin cytoskeleton regulatory pathway, thereby increasing the aggregation and stability of actin in OSCC [120]. Distal metastasis of malignant tumor cells involves complicated biological processes for which actin cytoskeleton motility and migration are critical [121]; therefore, the Khib-modified actin cytoskeleton regulatory pathway dynamically regulates the actin cytoskeleton, hence controlling and impacting OSCC metastasis. Additionally, the palmitoylation of EGFR regulated by Ras-related protein Rab-27A (RAB27A) via ZDHHC13 increased the proliferation, migration, and invasion of OSCC cells [122].

Consistent with Khib and palmitoylation in OSCC, the significance of Ksuc in thyroid carcinomas (TCs) was also highlighted. It was found that SDH participates in the modulation of Ksuc, thus promoting angiogenesis, glycolysis, and metastasis by enhancing gene expression, ultimately facilitating TC progression [123]. Furthermore, it was reported that individuals with SDHx variant carriers (SDHvar+) alone exhibited a higher prevalence of TC [124]. Additionally, thyroglobulin is traditionally considered a potential predictor in aiding the diagnosis of TC. Some scholars demonstrated that the noncovalent bonds of 19S thyroglobulin break and unfold after Ksuc, thus influencing T3 and T4 synthesis; other studies disclosed that extensive Ksuc of 12S thyroglobulin promote its self-cleavage, thus improving the interaction between hormones on the thyroid membrane and their specific receptors by enhancing the thyroglobulin affinity for membrane receptors. Moreover, the GDP-forming beta subunit of succinate-CoA ligase (SUCLG2) was recommended as a candidate biomarker to distinguish between follicular adenoma and follicular carcinoma [125]. These results suggest an association between Ksuc and TC progression.

**Fig. 2:**
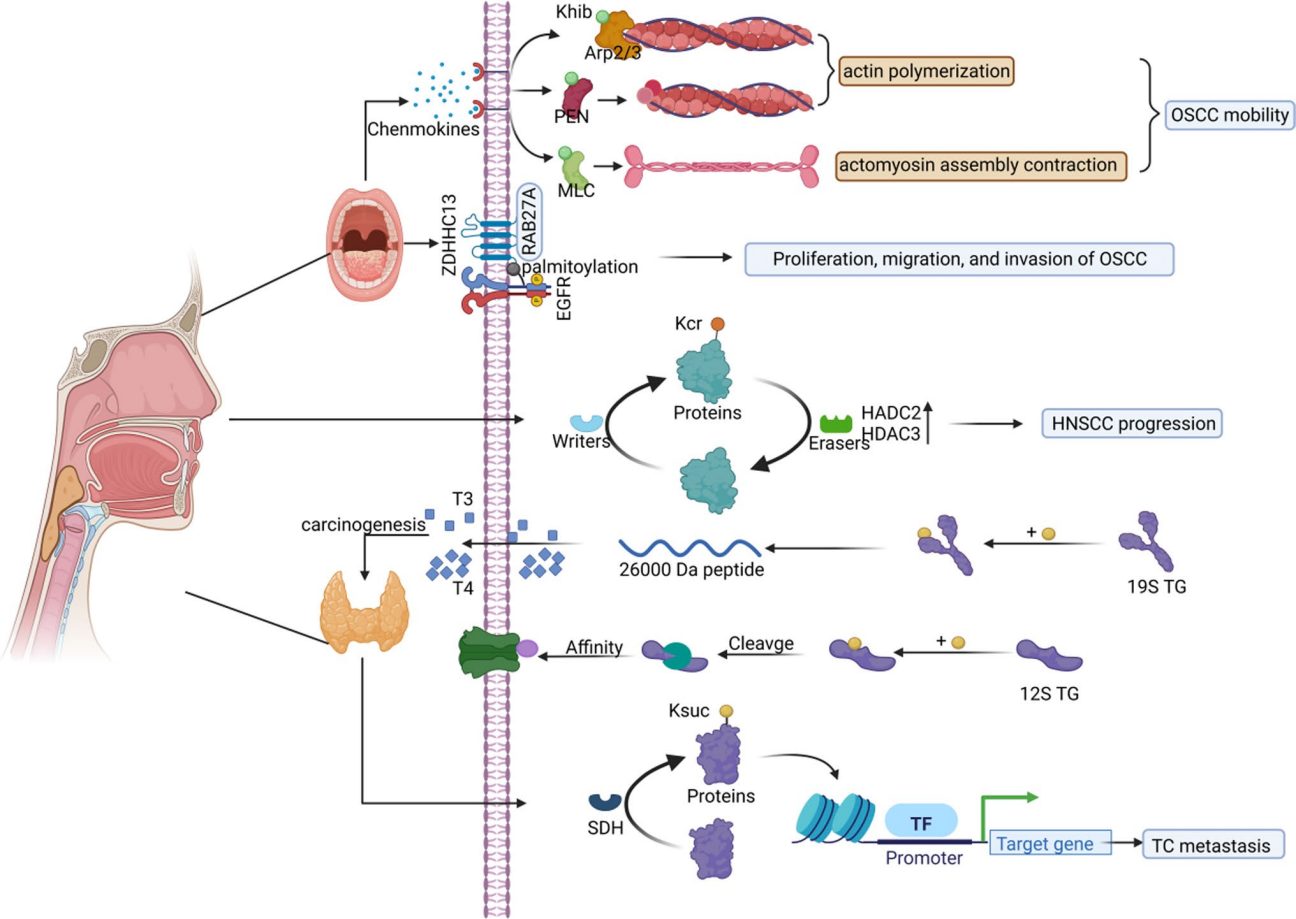
Novel acylations participate in head and neck cancer metastasis. In OSCC, Khib upregulation in multiple proteins are correlated with the actin cytoskeleton regulatory pathway, and thereby increasing the aggregation and stability of actin; EGFR palmitoylation promotes the proliferation, migration, and invasion of OSCC cells. In HNSCC, most Kcr regulators, such as DPF2, KAT2B, and HDAC2–3 are significantly dysregulated, which are closely correlated with the progression of HNSCC. In TC, Ksuc regulates the synthesis of T3 and T4, and improves the TG affinity for membrane receptors; SDH participates in the modulation of Ksuc, thus promoting TC metastasis as a result. (Created with BioRender.com.)

Moreover, it was also reported that most Kcr regulators, including DPF2, KAT2B, and HDAC2–3, were significantly dysregulated in patients with head and neck squamous cell carcinoma (HNSCC); this observation positively correlated with lymph-node metastasis, T stage, and histologic grade [126]. Specifically, KAT2B decreased in the advanced compared with the early T stage (*p* = 0.0016), disclosing the negative correlation between KAT2B and the T stage. HDAC2 was overexpressed in patients with versus without lymph-node metastasis (*p* = 0.035); thus, these data indicated that the progression of HNSCC is closely related to Kcr regulators, and it is vital to further classify the potential essential mechanisms of Kcr and these regulatory enzymes for HNSCC metastasis and progression. These results validate that head and neck cancer metastasis, including OSCC, TC, and HNSCC, exhibit a close, independent association with Khib, Ksuc, Kcr and palmitoylation (Fig. 2), thereby creating a new avenue for research regarding head and neck cancer progression.

### Breast cancer

**Fig. 3:**
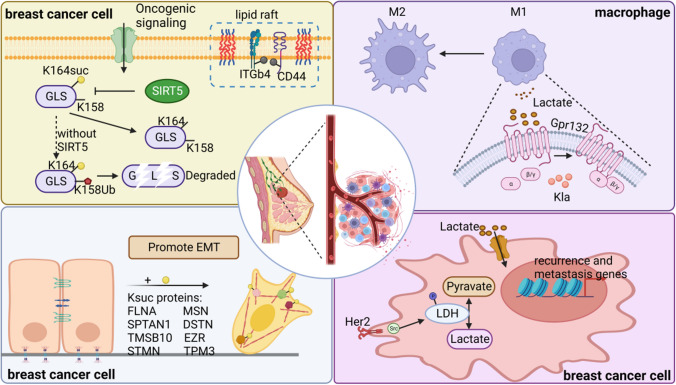
Novel acylations are critically involved in breast cancer metastasis. In response to oncogenic signaling, SIRT5 desuccinylates GLS at K164, and thereby block GLS ubiquitination at K158, promoting GLS from subsequent degradation. As a result, the invasion and proliferation of breast cancer cells are boosted. Furthermore, the regulators of actin cytoskeleton proteins are highly Ksuc-modified, modulating EMT of CSCs. Palmitoylation of CD44 and ITGβ4 modulate the affiliation of lipid raft, thus regulating the invasion of breast cancer cell. In addition, lactate and lactate-derived Kla are involved in regulating breast cancer metastasis. In one aspect, LDH in breast cancer cells mediates the interconversion of lactate and pyruvate, resulted in elevated cell invasion and reduced reactive oxygen species. In the other aspect, lactate and Kla promote the polarization of macrophages from the M1 to M2 phenotype, and ultimately progressing breast cancer metastasis. (Created with BioRender.com.)

Current evidence supporting the correlation between novel acylation and breast cancer metastasis is multidimensional (Fig. 3). The regulatory mechanism can be separated into three parts; first, it is recommended that novel acylation modulates breast cancer metastasis by directly regulating cell motility, e.g., in a study of MDA-MB-231 cancer stem cells (CSCs), actin cytoskeleton proteins, upstream regulators of EMT that participate in cytoskeleton organization, were highly Ksuc-modified. The authors also detected that lovastatin could prevent pulmonary metastasis and EMT by selectively targeting triple- negative breast cancer (TNBC)-derived CSCs via dysregulating cytoskeleton-related protein Ksuc [127]. Palmitoylation of CD44 and integrin β4 (ITGβ4) was reported to regulate invasive ability of breast cancer cell through modulating lipid raft affiliation [128, 129]. The second regulatory mechanism is indispensable for participation in metabolic reprogramming. Since the survival and invasion of TNBC are dependent on the levels of exogenous glutamine and mitochondrial enzyme glutaminase (GLS) activity, GLS desuccinylation at lysine residue K164 by SIRT5 was reported to block GLS ubiquitination at residue K158 and promote GLS from subsequent degradation [130]. SIRT5 inhibition significantly hampered breast cancer cell invasion and proliferation in high-glucose conditions [131], suppressed malignant transformation, and hindered lung metastasis in the MMTV-PyMT mouse model [132].

Given the complexity of lactate in regulating breast cancer metastasis, it can, therefore, be classified as the third regulatory mechanism. Macrophages exert prominent effects on cancer metastasis in the tumor microenvironment. One study demonstrated that lactate activates macrophage G protein-coupled receptor 132 to facilitate the phenotypic transformation to the alternatively activated M2 macrophage; this promotes cancer cell adhesion and invasion, ultimately progressing breast cancer lung metastasis [133]. Concomitantly, histone Kla derived from lactate drives the polarization of macrophages from the M1 to M2 phenotype [97]. Meanwhile, other studies claim that inhibition the activity of LDH, a catalytic enzyme that mediates the interconversion of lactate and pyruvate, resulted in attenuated cell invasion and elevated reactive oxygen species [134]. Treatment of MCF7 cells with lactate induced gene signatures associated with tumor recurrence and metastasis, and dramatically reduces overall survival [135]. Lactate acts as an oncometabolite, affecting the transcription of transcription factors (E2F1 and HIF1A), key oncogenes (RAS, MYC, and PI3KCA), cell cycle and proliferation genes (AKT1, ATM, CCND1, etc.), and tumor suppressors (BRCA1 an BRCA2) involved in breast cancer metastasis and progression [136]. Considering the high lactate level in the tumor microenvironment and the close correlation between lactate and Kla, it is believed that Kla may be a bridge that imperatively affects lactate, tumor metastasis, and patient outcomes. Encouragingly, research on HDACis in TNBC has achieved the desired results in suppressing metastasis. Expectedly, treating TNBC cells with entinostat (a specific HDACi) leads to increased expression of tumor suppressor proteins, including p53 and phosphatase and tensin homolog (PTEN), and antiangiogenic factor serpin family F member 1, in turn inhibiting angiogenesis and metastasis [137]. Likewise, a study revealed that HDAC inhibition increased HLA-DR and PD-L1 expression in tumor cells, and reducing the frequency of Treg cells thereby remodeling the tumor microenvironment [138]. The sole or combined application of HDACis and metabolism regulators might thus be a novel strategy to increase the antimetastatic efficiencyof breast cancer.

### Hepatocellular carcinoma (HCC)

**Fig. 4:**
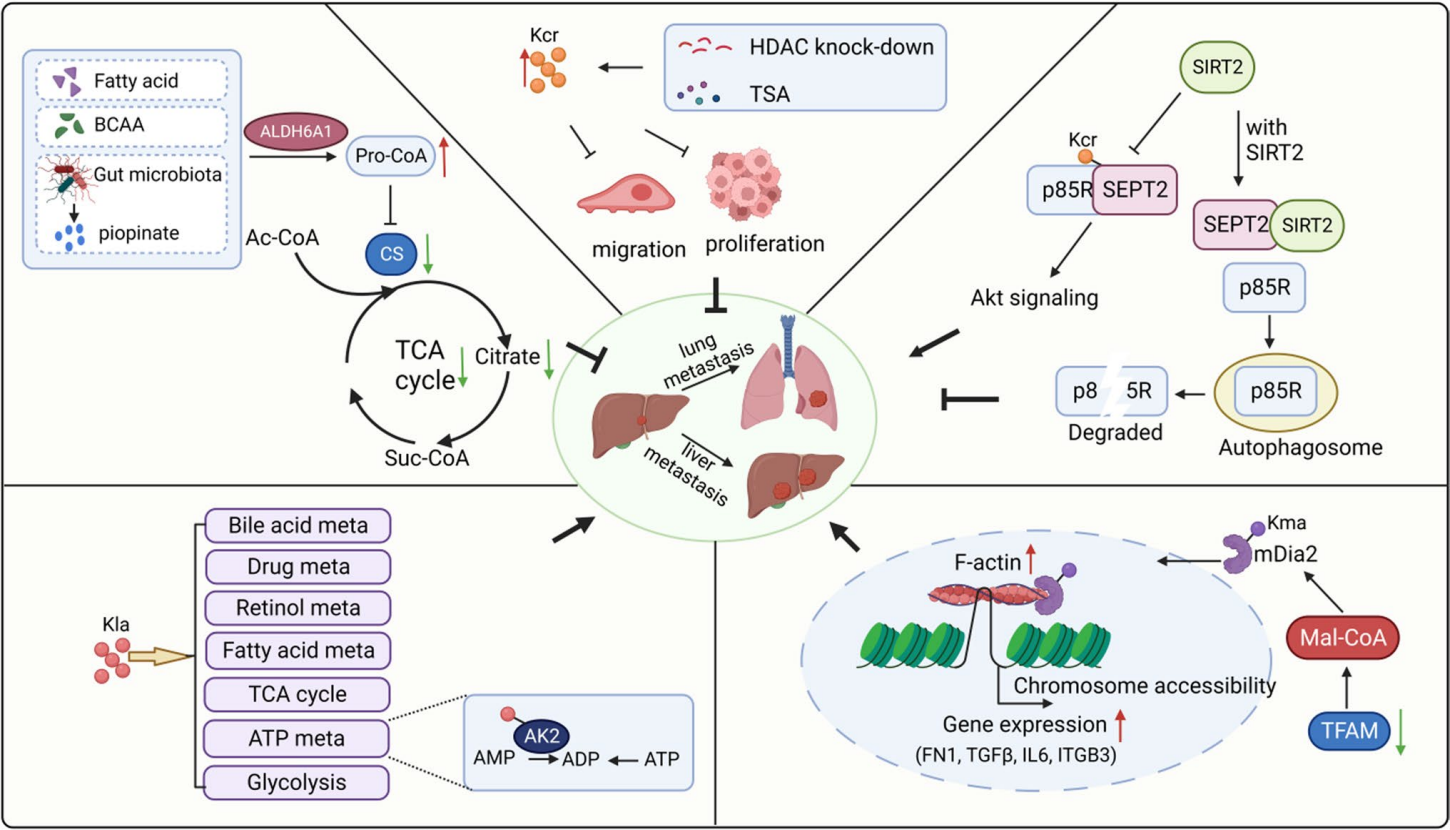
Novel acylations act as significant regulators in HCC metastasis. The Kcr of SEPT2 promotes the invasion of HCC cells through Akt signaling. The downregulation of TFAM increases the level of mal-CoA, and induces mDia2 Kmal, thereby facilitating the metastasis of HCC cells by promoting the expression of metastasis-promoting genes. Furthermore, Kla is involved in multiple metabolic-related pathways, thus promoting the proliferation and metastasis of HCC cells. In addition, ALDH6A1 upregulates the production of propionyl-CoA, thereby hampering CS activity. The CS with reduced activity suppresses the proliferation and migration of HCC cells through downregulating TCA cycle. Knockdown of HDACs or HDACi treatment improves the Kcr level, and thus limit hepatoma cell motility and proliferation. (Created with BioRender.com.)

Many studies have disclosed the role of various novel acylations as a double-sword for HCC metastasis (Fig. 4). Research has revealed that some novel acylations play a negative role in HCC metastasis, e.g., propionyl-CoA-derived propionyl-L-carnitine was characterized as an indicator to distinguish hepatitis from HCC [139]. In-depth investigations have documented a lower propionyl-CoA content in HCC than paired adjacent nontumor tissues; this content negatively correlated with the TNM stage and Edmondson grade of HCC tissues, suggesting a significant function in the development of HCC [140]. Mechanistically, aldehyde dehydrogenase 6 family member A1 (ALDH6A1) downregulation was shown to impair propionyl-CoA production in HCC, thereby inhibiting the activity of citrate synthase. Since citrate synthase is a critical mediator in the TCA cycle, which is closely linked to the energy source for HCC growth and invasion, perturbing ALDH6A1-elevated propionyl-CoA suppressed the proliferation and migration of HCC cells [140]. These observations indicate that propionyl-CoA and propionyl-CoA-related derivatives may be valuable and favorable biomarkers for HCC, as well as new diagnostic factors in HCC progression. Kcr expression was also downregulated in HCC, correlating with the TNM stage, while HDAC1 and HDAC3 simultaneous knockdown or HDACi trichostatin A (TSA) treatment improved the Kcr level, coincidently limiting hepatoma cell motility and proliferation [141]. These investigations suggest a negative correlation between Kpr and Kcr, as well as HCC metastasis and progression.

However, emerging evidence also highlights the positive relationship between novel acylation and HCC; it was documented that the Kcr of Septin 2 (SEPT2) promotes the invasion of HCC cells, with the SEPT2 in highly invasive HCC cells significantly hypercrotonylated; conversely, the K74 mutation of SEPT2 delayed its Kcr, leading to decreased GTPase activity of SEPT2 and impaired HCC metastasis in vitro and in vivo [142]. Previous research mentioned that mitochondrial transcription factor A (TFAM) downregulation in metastatic HCC induced Kma of mDia2 to promote its nuclear translocation and actin polymerization, thereby resulting in the high metastatic ability of HCC cells [143]. Moreover, Ksuc levels were suggested to predict the development of HCC; a high Ksuc level in HCC would result in a poor prognosis [144]. Simultaneously, a cohort analysis using hepatitis B virus-related HCC data showed that compared with Kac, Kla presents a unique and prevalent modification pattern beyond transcriptional regulation and histone proteins. The Kla of adenylate kinase 2 at K28 prohibits its activity, thus promoting the proliferation and metastasis of HCC cells [145]. Intriguingly, as the raw material of multiple acylations is provided by lipid metabolism (which is mainly processed in the liver), these acylations represent great potential as a therapeutic target for HCC metastasis. Moreover, according to the evidence mentioned above, the effect of novel acylations on HCC metastasis varies. Therefore, it is speculated that there is either competitive or synergistic crosstalk among different types of novel acylations, proposing the necessity of fully considering investigations of acylation-modified proteins and suggesting the significance of protein modification mapping.

### Gastrointestinal cancers

**Fig. 5:**
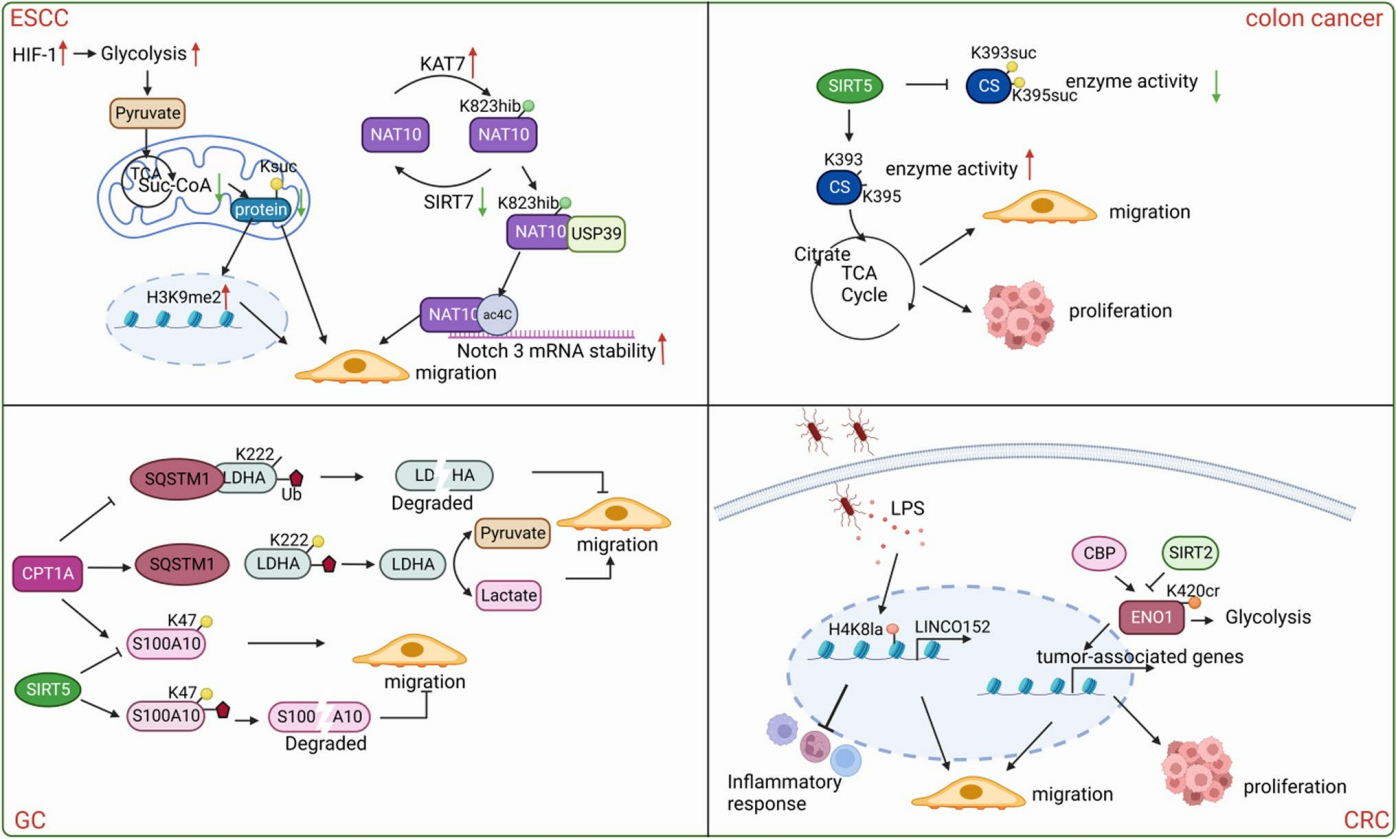
Novel acylations paly crucial roles in gastrointestinal cancers metastasis. In ESCC, decreased protein Ksuc and protein Ksuc-induced H3K9me2 enhancement in response to HIF-1 together promote the migration of ESCC cells; Khib-modified NAT10 increases the mRNA stability of NOTCH3 in an ac4C-modification dependent manner, thus driving ESCC metastasis. In colon cancer, SIRT5 desuccinylates CS and increases its catalytic activity in the TCA cycle, thereby promoting the proliferation and migration of colon cancer cells. In GC, CPT1A enhances the Ksuc of LDHA and S100A10, resulting in increased migration of GC cells, while SIRT5 decreases the Ksucc of S100A10. In CRC, enterobacterial induces H4K8la and drives the upregulation of LINC00152, thus facilitating the invasion and migration of CRC cells; elevated ENO1 Kcr promotes the migration and growth of CRC cells. (Created with BioRender.com.)

Gastrointestinal cancers mainly refer to esophageal, gastric, colorectal, pancreatic, and liver cancer. Here, we mainly discuss the first four cancers (excluding liver cancer). Similar to HCC, the effects of novel acylations in gastrointestinal cancers can be divided into two sections (Fig. 5). Some scholars proposed that specific novel acylations inhibit gastrointestinal cancer metastasis. They found that microbially-produced metabolic propionate-induced Kac and Kpr upregulates major histocompatibility complex class I polypeptide-related sequence A and B (MICA/B) expression in colon cancer cells, thereby inhibiting the development of colon cancer [146]. The Ksuc level dramatically declined in the malignantly transformed esophageal epithelial cell line, SHEEC; once the Ksuc is rescued, the malignant behaviors, including cell migration of esophageal squamous cell carcinoma (ESCC) cells, are inhibited [147]. The hypersuccinylation of citrate synthase at K393 and K395 by SIRT5 significantly decreased its catalytic activity in the TCA cycle and inhibited the proliferation and migration of colon cancer cells [148].

Contrastingly, numerous studies revealed an opposite relationship. In one example, S100A10 Ksuc was upregulated with the elevated expression of lysine succinyltransferase via CPT1A and decreased expression of desuccinylase via SIRT5, protecting S100A10 from ubiquitin-dependent proteolysis [149]. Furthermore, LDHA was found to be hypersuccinylated at K222 catalyzed by CPT1A in gastric cancer (GC) to promote GC cell proliferation, invasion, and migration. Differing from succinylated S100A10, LDHA Ksuc protects LDHA from lysosomal degradation by decreasing the interaction between ubiquitinated LDHA and sequestosome-1 (SQSTM1), with no effect on LDHA ubiquitination [150]. Additionally, the abundance of Kla was identified to predict prognosis in patients with gastric tumors; tumors were less lactylated in patients without than with lymph-node metastasis, and patients with Stage I–II gastric carcinoma had lower Kla levels than those with Stage IIIA–IIIB [105]. Moreover, Kla was found to drive the upregulation of LINC00152 induced by enterobacterial LPS in colorectal cancer (CRC) cells, thus facilitating the invasion and migration of CRC cells and providing an innovative perspective into how enterobacteria influence the progression of CRC [151]. Additionally, Kcr and Khib modifications were also verified to contribute to gastrointestinal cancer metastasis, with elevated ENO1 Kcr in human CRC tissues promoting CRC cell invasion, migration, and growth by increasing ENO1 activity and upregulating tumor-associated expression [152]. Inhibiting Khib using MG149, a selective Tip60 inhibitor, significantly reduced the invasion, migration, and proliferation of PC cells [153].

Another recent study revealed that compared with the corresponding parental cells, a specific elevation in Khib (not other PTMs) was detected in highly invasive and metastatic ESCC cell sublines [154]. Specifically, the nonhistone Khib modification at lysine residue K823 in RNA acetyltransferase enzyme N-acetyltransferase 10 (NAT10) reinforces its binding to ubiquitin-specific peptidase 39 (USP39), a deubiquitinase in protein degradation, leading to increased protein stability of NAT10. NAT10 was found to prolong the mRNA half-life of NOTCH3; consequently, Khib-modified NAT10 increased the mRNA stability of NOTCH3 in an N4-acetylcytidine (ac4C)-modification dependent manner, thus driving ESCC metastasis [154]. Therefore, NAT10 K823hib can be considered a predictor for the prognosis of patients with ESCC. Despite considerable advances regarding novel acylation in gastrointestinal cancers, further in-depth mechanism studies are essential; additionally, considering individual patient characteristics, a large sample cohort analysis is required. These findings suggest that the function of novel acylation in gastrointestinal cancer metastasis is complex and context-dependent.

### Glioma

**Fig. 6:**
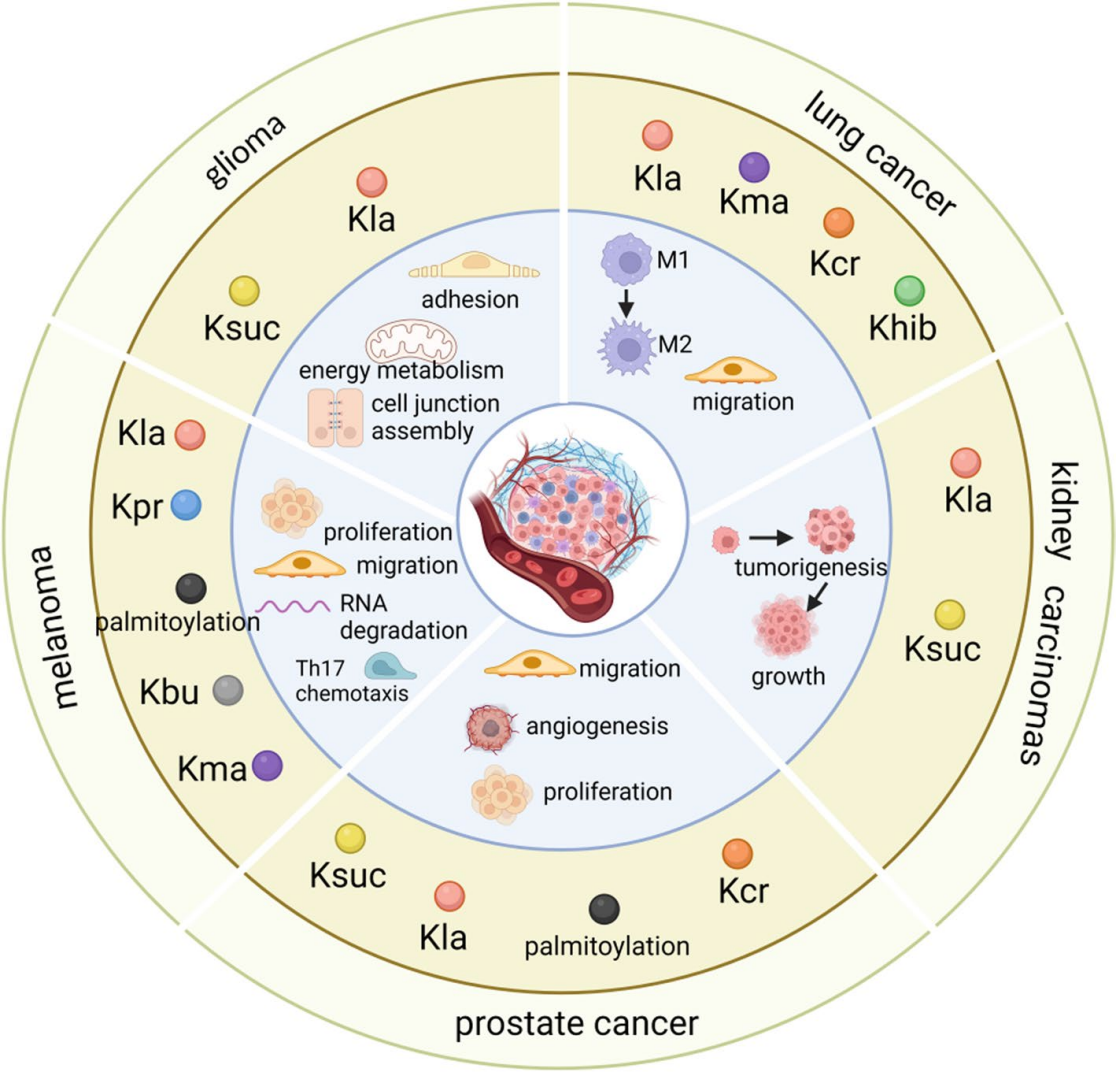
A summary of novel acylations for regulating caner metastasis in glioma, lung cancer, kidney carcinoma, PCa, and melanoma. In glioma, Ksuc regulates the malignancy of GBM; Kla might modulate cell junction assembly and actin cytoskeleton organization. In lung cancer, novel acylation-modulated M2 polarization and cell migration are involved in promoting lung cancer cells metastasis. In kidney carcinoma, Ksuc and Kla are proposed to participate in tumorigenesis and outgrowth, in addition to metastasis. In PCa, Kcr, Ksuc, palmitoylation and Kla are closely associated with the proliferation, invasion, or migration of PCa cells. In melanoma, Kpr, palmitoylation, Kbu, Kma or Kla promotes metastasis by regulating cell proliferation and migration, inducing RNA splicing, or mediating chemotaxis of Th17 cell. (Created with BioRender.com.)

Epigenetic abnormalities have long been considered a main driver for glioma malignancy. The regulatory function of Kac on glioma cell metastasis has been investigated by multiple researchers. HDAC1 was found to be overexpressed in GBM, as well as inhibit the expression of solute carrier family 30 member 3 (SLC30A3) by inducing the deacetylation of histone H3K27 of SLC30A3 around the super-enhancer region, thus hampering the outgrowth and metastasis of GBM cells [155]. In addition to HDAC1, Tip60, and HDAC4 were also revealed to be closely correlated with advanced tumor stage in glioma tissues, which regulate the invasion of GBM cells [156, 157]. Emerging investigations have gradually focused on the role of novel acylation modifications (excluding Kac) in glioma. It was reported that KAT2A binds with a stronger affinity to succinyl-CoA than acetyl-CoA in U251 GBM cells, demonstrating the innovative role of Ksuc in regulating GBM progression (Fig. 6) [53].

The occurrence of transgelin-2 (TAGLN2) dysregulation [158], which is highly expressed in glioma tissues and closely correlates with prognosis and tumor stage, is known to cause certain malignancies [159]. Interestingly, TAGLN2 was detected with 15.36-fold higher Ksuc at K40 in glioma endothelial cells (GECs) than in normal endothelial cells (NECs), significantly promoting the adhesion and metastasis of GECs. Furthermore, the hypersuccinylation of TAGLN2 at K40 also markedly promoted glioma angiogenesis and destroyed the integrity of GECs; this subsequently facilitated the migration of glioma cells, correlating with poor prognosis in patients with glioma [160]. A recent article reported that lactate was metabolized by GBM cells, acting as a modifier of histone H3K27ac that regulates mitochondrial energy metabolism, cell junction assembly, and actin cytoskeleton organization by promoting transcription (Fig. 6) [107]. However, it is necessary to consider the key role of Kla as the major effector of lactate in inducing lactylation during this process.

Considering the overlapping functions of various elements in Kac and novel acylation modifications, the necessary role of novel acylation modifications in glioma metastasis cannot be ruled out; still, there are limited studies regarding the association between glioma cell metastasis and novel acylation modifications.

### Lung cancer

Extensive investigations have provided insight into the potential functions of novel acylations in lung cancers (Fig. 6). First, Khib (not other PTMs) specifically increased in lung invasive cancer cells and tissues compared with corresponding parental cells [154]. When comparing small cell lung tissues with paired normal lung tissues from different patients, several tumor metastasis and tumor microenvironment regulators, such as caveolin-1, platelets, endothelial cell adhesion molecule 1, and stathmin 1 (STMN1), are modified by Kcr, underlying the broad spectrum of functions of Kcr-modified proteins in small cell lung cancer (SCLC). Additionally, the authors showed that crotonyl-CoA transferases, including KAT2A, KAT8, and p300, exhibit relatively higher expressions in SCLC, while only p300 had a positive overall survival in the Kaplan–Meier survival analysis [161]. Additionally, it is well known that M2-polarized tumor-associated macrophages are responsible for cancer progression and metastasis and promote the migration and invasion of lung cancer cells, resulting in decreased overall survival in tumor-bearing mice [162]. Lactate and histone Kla were reported to drive the expression of genes associated with M2 polarization [97]; therefore, Kla may play an indirect role in lung cancer metastasis and may serve as a potential therapeutic target for lung cancer.

Besides increasing cancer metastasis through M2 macrophages, lactate was also reported to directly modulate the migration of nonsmall cell lung cancer (NSCLC) cells. The migration abilities of NSCLC cell lines H1299 and A549 were inhibited by lactate [104]; thus, lactate may regulate lung cancer metastasis through multiple pathways. Furthermore, as a donor of Kma, malonyl-CoA was carboxylated from acetyl-CoA by key fatty acid metabolic enzymes, ACCs. Studies found that ACC2 underexpression positively correlated with clinical stage, regional lymph-node metastases, and tumor size in patients with lung adenocarcinoma, while ACC2 knockdown in HCC827 and A549 cells can facilitate the migration and proliferation of tumor cells [163, 164]. Moreover, HDAC1 expression increased in NSCLC cell lines and malignant tissue samples, which was associated with poor prognosis in NSCLC [165]. Considering the vital role of HDAC1 in Kcr, Kbhb, Khib, and Kla, its function in regulating the progression of lung cancer by mediating these acylations is acceptable. Finally, Cu/Zn superoxide dismutase was detected to be desuccinylated by SIRT5 and subsequently activated, thereby promoting the growth and metastasis of lung cancer cells [166, 167]. These observations demonstrate that many novel acylations play coordinated roles in modulating lung cancer metastasis; still, the detailed regulatory mechanisms remain to be further elucidated.

### Kidney carcinoma

Existing research on novel acylation modifications in kidney carcinoma metastasis mainly focuses on Ksuc and Kla (Fig. 6). Similar to its contribution to both colon and GC, the dysregulation of protein Ksuc in clear cell renal cell carcinoma (ccRCC) was significant. In total, 135 succinylated sites were identified in 102 proteins in ccRCC and adjacent normal tissues; specifically, SDH subunit A (SDHA) was highly desuccinylated at the K547 site in ccRCC, ultimately promoting ccRCC tumorigenesis [168]. SIRT5 was therefore proposed to be a potential therapeutic target for ccRCC based on the desuccinylation of SDHA and PDHA1 [168, 169]. Additionally, another study focused on underscoring the significance of histone Kla in boosting ccRCC metastasis and progression. In ccRCC, inactive von Hippel–Lindau positively correlated with histone Kla, indicating a poor prognosis in patients. Mechanically, histone Kla triggered by inactive von Hippel–Lindau stimulates the transcription of platelet-derived growth factor receptor β, whose signaling activates histone Kla and creates a positive feedback loop that promotes the progression of ccRCC. Thus, targeted correction of abnormal histone Kla inhibits the growth and metastasis of ccRCCs [170]. Collectively, these investigations may lay the foundation for further exploration of other novel acylation in kidney carcinoma metastasis.

### Prostate cancer (PCa)

Compared with their adjacent tissues, high levels of histone Kcr were observed in PCa tissues, positively correlating with the pathologic stages of tumors. BRD4, a member of the bromodomain and extraterminal domain family of proteins, can regulate the level of histone Kcr by regulating the expression of p300 and GCN5 in PCa cells. Decreasing histone Kcr using BRD4 inhibitors can alleviate the proliferation, invasion, and migration of PCa cells [171]. Analogously, high Ksuc levels were also detected in cancer tissues of patients with PCa compared with adjacent and normal prostate tissues [172]. Nevertheless, for patients with PCa and Gleason grades III–IV, the Ksuc modification was highly overexpressed, demonstrating its close relationship with the degree of invasion, metastasis, malignancy, and prognosis of PCa [173]. Nonhistone Ksuc was also shown to contribute to PCa metastasis; e.g., SIRT5 reduction-derived LDHA-K118suc significantly increased the activity of LDH and enhanced the migration and invasion of PCa cells in patients with PCa [174]. The Ksuc of C-terminal binding protein 1 (CTBP1) K46 and K280, induced by KAT2A, inhibits the suppressive activity of CTBP1 acting on CDH1 transcription; it was thus identified as an oncogene regulating the metastasis and viability of PCa cells [175]. Moreover, the Kla of HIF1α was reported to boost KIAA1199 transcription in PCa tissues and increase the expression of VE-cadherin, VEGFA, and phosphorylated EphA2, therefore promoting angiogenesis and vasculogenic mimicry [163]. KAI1/CD82 is a tumor metastasis suppressor, and the palmitoylation of KAI1/CD82 effectively blocked its motility-inhibitory activity [176]. Together, novel acylations in histones and nonhistones synergistically regulate PCa cell metastasis (Fig. 6).

### Melanoma

Multiple studies have proposed that Kla functions as a double-edged sword in melanoma progression. In ocular melanoma cells, aerobic glycolysis-derived histone Kla elevation increased the expression of YTH (YT521-B homology) domain 2 (YTHDF2), which binds to the m6A sites of TP53 and PER1 mRNAs for RNA degradation, thereby contributing to the aggressiveness of ocular melanoma cells [177]. Another study found that lactate treatment (20 mM) significantly decreased the migration and proliferation of cells, acting by switching cell metabolism toward oxidative phosphorylation in uveal melanoma, which rewrites the cells to a quiescent phenotype [178]. These findings demonstrate the complexity of Kla and the metabolic regulation of melanoma progression. Additionally, it was revealed that propionate and butyrate (produced by gut microbiota after probiotic supplementation) facilitate the secretion of chemokine (C–C motif) ligand 20 via lung endothelial cells and subsequently act as a chemokine to recruit T helper 17 cells to the lungs. This process attenuates the number of metastatic foci of melanoma in tumor-bearing mice [179]. Furthermore, acetyl-CoA and malonyl-CoA, the donors of Kac and Kma, respectively, produce endogenous fatty acids mediated by FASN. FASN was reported to promote spontaneous lymphatic metastasis in a murine model of B16F10 melanoma [180]. Wnt5a-induced depalmitoylation of melanoma cell adhesion molecules (MCAM) promoted melanoma cell invasion ability [181]. It was also reported that glutaryl-CoA dehydrogenase, which controls protein Kglu and has significant functions in lysine metabolism, was identified as an irreplaceable element that limits apoptotic signaling by controlling nuclear factor E2-related factor 2 (NRF2) glutarylation in melanoma [182]. These studies show that whether novel acylations function as a tumor promoter or suppressor is determined by the predominant regulatory pathway (Fig. 6).

## Clinical significance

**Fig. 7:**
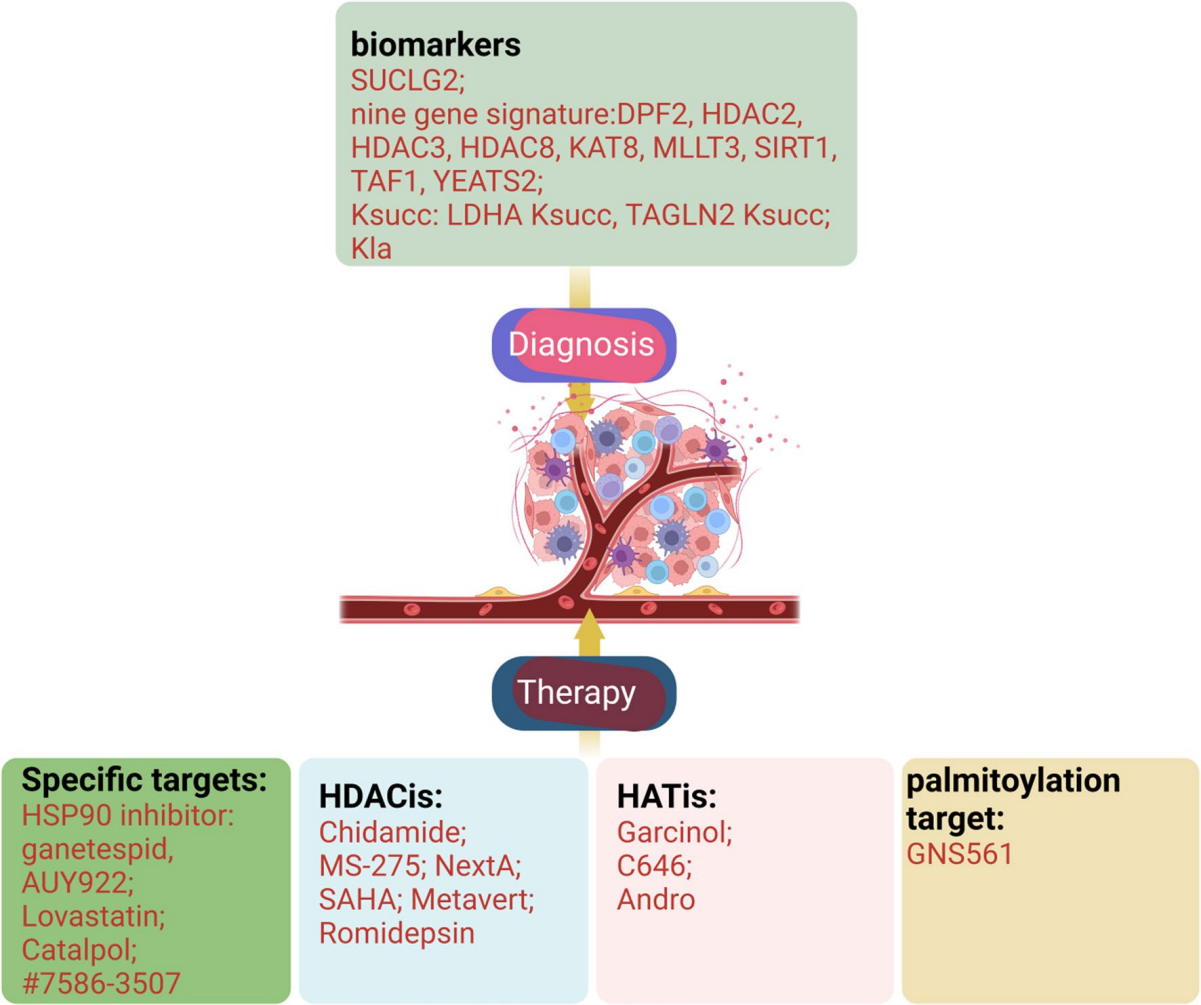
Clinical significance of novel acylations in tumor metastasis. Diagnostic biomarkers that have been reported as predictors of tumor metastasis progression are listed in the upper. Inhibitors that have achieved ideal effects in preclinical are listed in the below. (Created with BioRender.com.)

Considering the close association between acylation and tumor metastasis, it is possible to regard novel acylations as a predictor of tumor metastasis progression. Encouragingly, numerous preclinical tests have been performed, achieving positive results (Fig. 7). Furthermore, multiple preclinical trials reveal that the inhibitors of regulatory enzymes of acylation have significant effects on suppressing tumor metastasis (Fig. 7); however, their mechanisms remain to be further explained. Therefore, the regulatory elements that participate in and molecules that undergo acylation may become potential therapeutic targets (Table 2).

**Table 2:** Drugs that target acylation-related molecules

Targets	Drugs	Function in metastasis	Cancer types	References
Specific targets	HSP90 inhibitor (AUY922 and ganetespib	Antitumor activity by affecting Ksuc modification	Bladder cancer	[191]
	Lovastatin	Inhibit stemness properties of CSCs through Ksuc of proteins involved in cytoskeleton; inhibit Metastasis	Breast cancer	[128, 194, 195]
	Catalpol	Increase the Kac, Khib, and Kla	A variety of cancers	[196–198]
	#7586-3507	Inhibit the invasion of ESCC cells	ESCC	[155]
HDAC	Tucidinostat (Chidamide)	Down-regulate ERK signaling and decrease MMP2	Nasopharyngeal carcinoma	[201]
	MS-275	Increase vascular permeability	Melanoma	[202]
	NextA	Improve immune response and diminish aggressiveness	Breast cancer	[203–205]
	SAHA + TDZD	Inhibit EMT	PDAC	[197]
	Metavert	Reduce tumor size, prevented metastasis, and prolong survival	PDAC	[211]
	Romidepsin + paclitaxel	Eradicate the metastatic lesions and reduce tumor size	Breast cancer	[213]
	Romidepsin + erlotinib	Enhances the sensitivity of nonsmall cell lung cancer cells to erlotinib	Nonsmall cell lung cancer	[215]
	Romidepsin + docetaxel	Increases cytotoxicity of docetaxel	Prostate cancer	[216]
	MS-275 + AZA	Induce DNA damage, loss cell viability, promote apoptosis, and decrease cell migration	Esophageal cancer	[217]
HAT	Garcinol	inhibit cancer cells migration and invasion	Esophageal cancer	[218]
	C646 + gemcitabine	Increase gemcitabine-induced cell apoptosis	Pancreatic cancer	[219]
	Andrographolide	Inhibit NF-κB activation; suppress angiogenesis	Breast cancer	[220]
Palmitoylation	GNS561	Modulate lysosomal function	Hepatocellular carcinoma	[222]

### Diagnostic biomarkers

Considerable evidence suggests novel acylations and their regulatory elements as diagnostic biomarkers of tumor progression and metastasis. SUCLG2, a subunit of succinate-CoA ligase, participates in generating succinate from α-ketoglutarate in the TCA cycle [125, 183]. SUCLG2 is estimated to be a biomarker candidate that can effectively differentiate between follicular adenoma and carcinoma with 75% sensitivity and 80% specificity, depending on a specific cutoff score extrapolated from the intensity and percentage of immunohistochemistry (IHC) staining [125]. Based on Kcr regulators, a nine-gene signature, including DPF2, HDAC2, and HDAC3, was established and validated as an independent factor for prognosis in patients with HNSCC; this could effectively distinguish patients into low- and high-risk groups depending on the difference in OS using the GEO-HNSCC dataset (*p* = 0.023) [126]. Univariate analysis of training cohorts revealed that the risk characteristics obtained from the nine-gene regulators were significantly associated with OS (*p* < 0.001, HR 4.175, 95% CI 2.573–6.776); the nine-gene risk assessment from the multivariate analysis further verified their potential as independent prognostic factors (*p* < 0.001, HR 3.500, 95% CI 2.178–5.625) [126]. Additionally, the TNM stage of multiple types of cancers directly correlated with novel acylation; e.g., liver cancer tumor tissues show higher succinylation levels than adjacent tissues (*p* < 0.001), and according to the univariate and multivariate analyses, the OS closely correlates with the Ksuc intensity (IHC score; univariate analysis: *p* = 0.0073, HR 1.2, 95% CI 1.1–1.4; multivariate analysis: *p* = 0.00023, HR 1.29, 95% CI 1.13–1.48) [144]. Concomitantly, the protein Ksuc landscape in GC tissues differs significantly from that of adjacent normal tissues; specifically, the Ksuc of LDHA in GC tissues is highly overexpressed (1.42-fold higher than adjacent normal tissues), and the LDHA Ksuc differs significantly regarding pathological grade and clinical stages [150]. Furthermore, the TAGLN2 Ksuc level in GECs is 15.36-fold higher than in NECs, thus promoting angiogenesis in glioma [160]. Ksuc overexpression can predict a poor prognosis and a high degree of metastasis; therefore, modification may become a significant prognostic biomarker in glioma, HCC, and GC.

Recently, Kla was found to be more enriched in gastric tumors than adjacent tissues, and the level was higher in poorly differentiated than moderately differentiated tumors. Furthermore, the Kla level is positively related to the TNM stage, lymphatic metastasis, and OS of patients with GC [105]. Therefore, many types of acylations and acylation-associated regulatory elements have demonstrated their potential as independent prognostic indicators, and further investigation is necessary to verify their accuracy and efficiency in prospective and multicenter clinical trials.

### Therapeutic targets

#### Specific targets

Currently, the remarkable effect of multiple inhibitors targeting the upstream and downstream pathways of novel acylation may inspire the therapeutic intervention strategy in the clinic. Heat shock protein 90 (HSP90) is a molecular chaperone that participates in the activation of disparate client proteins. It is highly expressed in multiple types of tumors, and its overexpression correlates with the poor prognosis of NSCLC and breast cancer [184, 185], facilitating the metastasis of CRC and HCC [186, 187]. HSP90 inhibition has achieved promising antitumor effects in multiple animal cancer models; e.g., HSP90 inhibitors, including ganetespid, and AUY922, significantly suppress the progression of bladder cancer by affecting several types of core histone acylations, including propionylation, butyrylation, succinylation, and 2-hydroxyisobutyrylation in bladder cancer [188]. Additionally, the application of lovastatin in antitumor therapy has gradually attracted attention over the last few decades [189].

Clinically, lovastatin, a natural statin derived from *Monascus*-fermented rice or diostilbene and found in high concentrations in oyster mushrooms, has been intensively utilized to prevent and treat hyperlipidemia [190]. It was innovatively reported that lovastatin treatment could significantly suppress cancer cell invasion and metastasis, as well as induce cell apoptosis both in vitro and in vivo [191, 192]. Additionally, lovastatin was recently demonstrated to limit the stemness conversion of TNBC cell lines by succinylating the proteins involved in cytoskeleton pathways and inhibiting liver metastasis in breast cancer [127]. Moreover, Catalpol, an extract separated from the traditional Chinese medicinal plant *Rehmannia glutinosa,* has therapeutic effects on multiple types of cancers, including nonsmall-cell lung cancer, colon cancer, and GC [193–195]. Catalpol administration reduced the viability of MCF-7 cells and increased the levels of lactylation, 2-hydroxyisobutyrylation, and acetylation; conversely, the levels of malonylation, succinylation and phosphorylation dramatically declined in breast cancer tumors [196]. Recently, a small-molecule compound (#7586-3507) was found to exert dose-dependent anti-invasion effects on ESCC cells by directly targeting the Khib site in NAT10; 5 μM dramatically decreased the invasion rate to 10–20% [154]. These results further verify the potential of PTMs-targeting diagnostics and therapeutics.

#### HDACis

Four classes of HDACs comprise the HDAC family: class I includes HDACs 1–3 and HDAC8; class II includes HDACs 4–7 and HDACs 9–10; class III is the SIRT family; and class IV includes HDAC11. HDACis have long been used as anticancer drugs; tucidinostat (chidamide) was approved for the treatment of breast cancer and peripheral T-cell lymphoma by the National Medical Product Administration (NMPA), which is an orally bioavailable and potent class I HDACi [197]. Targeting DNTTIP1/HDAC1 using chidamide disrupts the ERK signaling pathways and decreases MMP2 expression by upregulating dual specificity phosphatase 2 (DUSP2); this inhibits the migration, invasion, and proliferation of nasopharyngeal carcinoma cells [198]. Notably, neuroblastoma is one of the most common extracranial solid tumors; there are limited therapeutic interventions, and studies demonstrate that MS-275 administration dramatically inhibits the expression of Robo4 in endothelial cells by suppressing HDAC1–3, thereby increasing vascular permeability [199]. Likewise, Nexturastat A was reported to improve the immune checkpoint blockade of antitumor immune response and diminish the aggressiveness of breast cancer cells with minimal cytotoxic effects by specifically targeting HDAC6 in breast cancer [200–202]. Nevertheless, overwhelming evidence suggests a low HDACi efficacy in cancer monotherapy; thus, a combination therapy strategy was gradually proposed. For instance, suberoylanilide hydroxamic acid (SAHA), an epigenetic HDACi drug targeting class I/II HDACs, was clinically approved for cutaneous T-cell lymphoma [203]. This confirmed that SAHA exhibited promising clinical benefits in neuroblastoma by regulating histone Kac and Kbu [204]. Of particular interest, Tideglusib (TDZD) treatment was reported to inactivate NF-κB and reduce the tumor burden in PDAC by inhibiting glycogen synthase kinase-3 beta, a commonly expressed serine/threonine kinase involved in metabolism, tumorigenesis, and neurological disorders [205, 206]. However, TDZD administration can also trigger EMT, potentially causing tumor invasion and metastasis as a side effect [207]. Interestingly, studies showed that combined treatment with small doses of SAHA and TDZD exhibited an additive effect, preventing cancer cell survival and inhibiting EMT. Due to the validity of this combination, a novel molecule, Metavert, was recently designed with reference to the SAHA and TDZD structures; its administration dramatically decreased tumor size, suppressed metastasis, and significantly prolonged the survival of mice with aggressive PDAC [208]. Romidepsin is an antineoplastic drug targeting class I HDACs. It was approved for application in peripheral T-cells, and refractory or relapsed cutaneous lymphomas [209]. Combined treatment with romidisin and paclitaxel effectively eradicated the metastatic lesions and reduced the size of primary tumors [210]. Additionally, romidepsin was found to collaboratively increase the sensitivity of NSCLC cells to erlotinib and enhance the cytotoxicity of docetaxel in preclinical models of androgen-independent prostate cancer [211–213]. A comparative analysis focusing on the combined effect of treatment with both HDACis (including SAHA, romidepsin, and MS-275) and DNA methyltransferase inhibitors (including Decitabine and Azacitidine) was performed in ESCC and esophageal adenocarcinoma. Data showed that combined treatment with MS-275 and Azacitidine specifically targeted esophageal cancer cell lines, not normal esophageal epithelium cell lines, by decreasing cell viability, inducing DNA damage and cell apoptosis, and decreasing cell migration. SAHA combined with FK228 treatment decreased the viability of cells without selectivity [214]; therefore, the combination of agents with various targets may provide more ideal clinical therapeutic effects.

#### HAT inhibitors (HATis)

HATs were also found to be potential drug targets for malignant tumors. The identification of small-molecule inhibitors for p300/CBP and PCAF HAT domains appeals to pharmacists looking to provide new routes for anticancer treatment. Garcinol, an effective inhibitor targeting p300 and PCAF domains, was confirmed to inhibit the invasion and migration of esophageal cancer cells in a dose-dependent manner [215]. Another report showed that treatment with C646, a specific p300 inhibitor, reduced histone acetylation and increased the sensitivity of cells to gemcitabine to induce cell apoptosis, thereby enhancing the cytotoxicity of gemcitabine against pancreatic cancer cells [216]. Andrographolide, isolated and identified from *Andrographis paniculate,* was reported to have multiple biological functions with effective anticancer properties. In humans, andrographolide was detected to inhibit NF-κB activation induced by COX-2 by targeting p300 for breast cancer treatment; it was also reported to suppress VEGF-derived endothelial cell motility by impairing cofilin activity and the formation of stress fiber, thus suppressing breast cancer angiogenesis [217]. These findings indicate that p300/CBP and PCAF may be potential targets for epigenetic therapies, underlying the prospect of simultaneously controlling tumor progression. The clinical translation of these inhibitors has a long way to go, and whether other HAT inhibitors, like p300/CBP bromodomain (CCS1477) and HAT (MC1626) inhibitors, are effective for suppressing metastasis remains to be elucidated.

#### Inhibitors targeting palmitoylation

Due to the difference in catalytic enzymes between palmitoylation and the other ten novel acylation forms, the inhibitors targeting palmitoylation are promising alternatives. The enzyme activity inhibitors or RNAi targeting PATs or APTs were regularly utilized to hamper the function of the enzymes. In fact, several studies have revealed that the inhibition of ZDHHCs or PPTs can block tumor development and invasion. Knockdown of DHHC5 or APT1 significantly decreased the colony formation, proliferation, and invasion abilities of NSCLC cells [108, 218]. GNS561, a clinical-stage inhibitor specifically targeting PPT1, effectively impaired the progression of hepatocellular carcinoma [219]. Though RNAi or inhibitors targeting PATs or APTs, significant advancements have been made in antimetastatic potential; however, their clinical application for inhibiting tumor metastasis is still in its infancy.

## Discussion

Many prominent investigations focused on the role of differentially expressed proteins in various biological functions. Protein PTM leads to increased proteome complexity; knowledge regarding protein functionality and diversity has thus further expanded, especially with the discovery of novel acylations dependent on advances in MS, proteomic technologies, and bioinformatics. Studies of these novel acylations provide new perspectives for determining transcriptional regulation, protein structure, subcellular translocalization, and protein interactions and offer new potential therapeutic strategies for the treatment of incurable diseases. Reversible lysine acylations and their regulatory elements play a significant role in tumor metastasis; some acylations exhibit potential as independent prognostic indicators, and some inhibitors involved in novel acylation processes have achieved encouraging efficacy. This review summarizes 11 types of novel acylations, supplemented with newly discovered regulatory enzymes; importantly, it emphasizes their roles in tumor metastasis and clinical potential.

Novel acylations are important epigenetic mechanisms that play key roles in the regulation of cell function by targeting both histones and nonhistones. Among the discovered classical HATs and HDACs, many were identified to mediate multiple acylation modification pathways simultaneously; while tremendous progress has been made, there still remain challenges and limitations. In spite of these encouraging achievements, the functional diversity of specific acylation modifications and their regulatory complexity via enzymatic or nonenzymatic mechanisms determine the difficulty of research. Therefore, their diverse regulatory processes in tumor metastasis could not be thoroughly figured out. For example, SIRT5-mediated GLS desuccinylation promoted the invasion of breast cancer cell by hampering the ubiquitination-dependent GLS degradation, while the deacetylation of GLS was also reported to contribute to the progression of various carcinomas [220–222]. Hence, it is necessary to comprehensively analyze the coordinated contribution of these intertwined modifications. Furthermore, how nonspecific HATs or HDACs specifically modulate one type of acylation and how the regulatory function selectivity of a particular acylation is controlled remain unknown. P300 was identified as a writer that mediates multiple acylations, including acetylation, propionylation, butyrylation, crotonylation, 2-hydroxyisobutyrylation, benzoylation, and lactylation. It is theoretically speculated that these acylation modifications would change simultaneously in response to p300; however, recent studies have shown that p300 exhibits modification selectivity and can only specifically regulate the occurrence of one or two acylation modifications under specific physiological or pathological conditions. Some reasons may be that p300 exhibits context-dependency for the acyltransferase activity of a particular modification, or that its acyltransferase activity differs in different organs, cells, or organelles; that the relative concentrations of different acyl-CoA forms in the cytoplasmic and nuclear pools determine the type of acylation; or that the regulatory mechanism of p300 for all acylation modifications lacks a systematic interpretation. Therefore, the exact regulatory mechanism balancing the various acylations remains to be further elucidated.

Notably, various acylation modifications play coordinating roles in regulating gene expression and metabolic processes. Given the intertwined metabolic sources of lysine acylation, it is unsurprising that these acylations crosstalk and share similar features in metabolism. Nevertheless, although all acylations were found in histone, and histone acylation represents an opportunity to regulate chromatin activation and gene translation, how the various acylations regulate different gene expressions remains unknown. Diverse acylations were demonstrated to mark active regulatory elements differently; e.g., histone butyrylation mainly regulates gene expression related to adipogenesis and lipid metabolism; H3K9bhb is implicated in active gene expression associated with metabolic pathways; and homeostatic gene translation is prompted by histone lactylation in response to inflammatory stimulation. During spermatogenesis, histone butyrylation, crotonylation, glutarylation, 2-hydroxyisobutyrylation, and acetylation coordinately contribute to gene expression in a stage-specific fashion. Some acylations, such as Kglu, were reported without selectivity, leading to an overall upregulation of transcription. Notably, acylation-induced gene activation was hypothesized to be signal-dependent and metabolically induced; the signal pathways are switched on/off in response to specific stimulations, and variations in the proportional mixture of distinct acylations determine their gene expression outcomes. We, therefore, considered the likelihood of more coordinated and precise regulatory mechanisms during differential histone acylation.

Based on the close correlation between novel acylation modifications and tumor metastasis, there is important clinical significance to the research on novel acylations and their modifying enzymes for tumor metastasis. On the one hand, acylations can be potential diagnostic biomarkers to predict the progression of tumor metastasis; on the other hand, targeting these acylations or their regulatory elements is expected to become an effective weapon for the prevention of malignant tumor metastasis by comprehensively exploring the regulatory mechanisms of novel acylation. Indeed, many inhibitors have exhibited encouraging targeted therapeutic effects in clinic. Given the necessary role of KAT/KDAC-mediated reversible lysine acylation in tumor metastasis, HDACis and HATis may become effective agents for the treatment and prevention of tumor metastasis. Detailed, large-scale investigations have focused on the inhibitory effectiveness of HDACis and HATis in tumor metastasis; however, although site-specific or other acylation modifications have specific functions during tumor metastasis, most reported HDACis and HATis have a broad range of targets. Many acylations share common HDACs and HATs: SAHA can both inhibit class I and II HDACs, while romidepsin predominantly suppresses class I HDACs. Some acylations clearly exhibit pro-metastatic functions, while others inhibit tumor metastasis; thus, HDACi treatment may simultaneously affect multiple acylations, resulting in unpredictable consequences. Additionally, different HDACs were reported to have differential functions: class I tends to induce cell proliferation and suppress cell differentiation and apoptosis [209]; class II promotes tumor angiogenesis [209]; class III sirtuins have diverse functions in multiple types of cancers [223]. HDACis were therefore elucidated to target various cell signaling pathways that might lead to pro- and anticancer activities and induce various cell reactions and clinical therapeutic outcomes.

As complex functional mechanisms, the cell type-specific effects of each inhibitor remain to be further considered. Current drug-targeted delivery strategies have attracted increasing clinical attention owing to their characteristics of achieving better biodistribution and compatibility, immunogenicity, therapeutic load, and drug detectability. Additionally, nanocarrier system (e.g., liposomes, hydrogel, micelles, inorganic nanomaterials, and nanocapsules)-dependent targeted delivery of drugs has made excellent progress in inhibiting tumor metastasis, e.g., nanoparticle-dependent drug delivery-targeting lymph nodes effectively relieve the lymphatic metastasis of tumors [144]. Therefore, the drug-targeted delivery system may highlight a new avenue for solving the nonspecificity inhibitors. Collectively, identifying isoform-specific HDACis with clear and uncontaminated molecular targets to promote the personalization of cancer treatment is of utmost importance.

## Conclusion

In summary, tremendous progress has been made regarding the role of novel acylation modifications in tumor metastasis. A better understanding of these modifications and their regulators may help identify novel therapeutic targets and diagnostic biomarkers for tumor metastasis.

## Data Availability

Not applicable.
